# Unified transmission electron microscopy with the glovebox integrated system for investigating air-sensitive two-dimensional quantum materials

**DOI:** 10.1016/j.xinn.2024.100751

**Published:** 2025-01-06

**Authors:** Qishuo Yang, Xingxing Li, Ludan Zhao, Gang Wang, Zenglong Guo, Kangdi Niu, Shaolong Jiang, Fuchen Hou, Junhao Lin

**Affiliations:** 1 Department of Physics and Guangdong Basic Research Center of Excellence for Quantum Science, Southern University of Science and Technology (SUSTech), Shenzhen 518055, China; 2 School of Mechanical and Mining Engineering, The University of Queensland, Brisbane, QLD 4072, Australia; 3 Quantum Science Center of Guangdong-Hong Kong-Macao Greater Bay Area (Guangdong), Shenzhen 518045, China; 4 Guangdong Provincial Key Laboratory of Advanced Thermoelectric Materials and Device Physics, Southern University of Science and Technology, Shenzhen 518055, China

## Abstract

Transmission electron microscopy (TEM) is an indispensable tool for elucidating the intrinsic atomic structures of materials and provides deep insights into defect dynamics, phase transitions, and nanoscale structural details. While numerous intriguing physical properties have been revealed in recently discovered two-dimensional (2D) quantum materials, many exhibit significant sensitivity to water and oxygen under ambient conditions. This inherent instability complicates sample preparation for TEM analysis and hinders accurate property measurements. This review highlights recent technical advancements to preserve the intrinsic structures of water- and oxygen-sensitive 2D materials for atomic-scale characterizations. A critical development discussed in this review is implementing an inert gas-protected glovebox integrated system (GIS) designed specifically for TEM experiments. In addition, this review emphasizes air-sensitive materials such as 2D transition metal dichalcogenides, transition metal dihalides and trihalides, and low-dimensional magnetic materials, demonstrating breakthroughs in overcoming their environmental sensitivity. Furthermore, the progress in TEM characterization enabled by the GIS is analyzed to provide a comprehensive overview of state-of-the-art methodologies in this rapidly advancing field.

## Public summary

• The current research focuses on water- and oxygen-sensitive two-dimensional materials with excellent physicochemical properties.
• A glovebox-integrated system was developed, which provides a new platform for investigating the structure and properties of water-sensitive two-dimensional materials.
• The glovebox-integrated system is ideal for characterizing transition metal dichalcogenides, two-dimensional dihalides, and other low-dimensional sensitive materials.

## Graphical abstract

**Figure:**
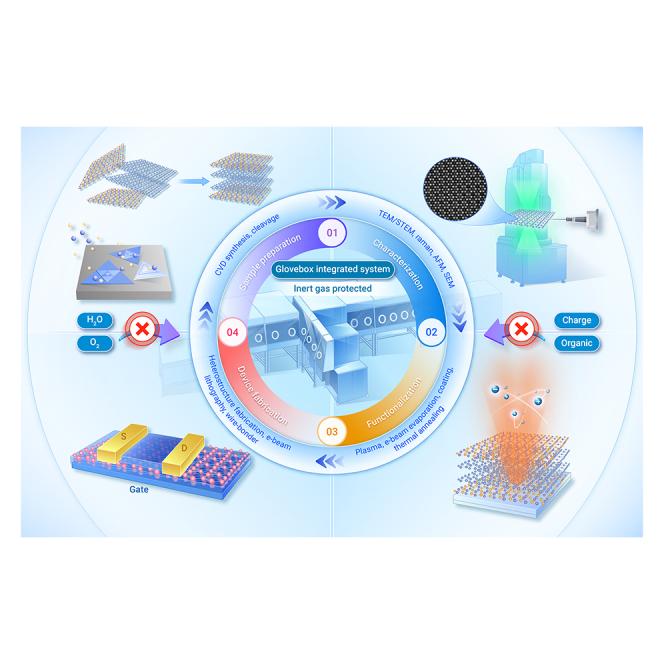

## Introduction

Two-dimensional (2D) materials with a diverse family, formed through continuous scientific exploration, exhibit unique properties attributed to their atomic-level thickness and unique planar structure. In recent years, thus, 2D materials have emerged as a focal point of research in condensed matter physics, materials science, and nanoelectronics.^1^^,^^2^^,^^3^^,^^4^ 2D material was first discovered in 2004 when Geim’s group at the University of Manchester successfully isolated a single atomic graphene layer from graphite.^1^ When looking for high-performance lithium-ion anode materials, Gogotsi, Barsoum, and co-workers accidentally revealed a series of 2D layered materials such as Ti_3_AlC_2_, Ti_2_C, and Ta_4_C_3_ in 2011 and named them MXenes (M is transition metal, X is C or N).^5^ Simultaneously, researchers succeeded in isolating monolayers from a class of compounds known as transition metal dichalcogenides (TMDCs) that were discovered in the early 20th century, expanding the range of 2D materials. In organic materials, metal-organic frameworks and covalent organic frameworks have also been successfully separated into ultrathin 2D layered structures.^6^^,^^7^^,^^8^

**Figure 1:**
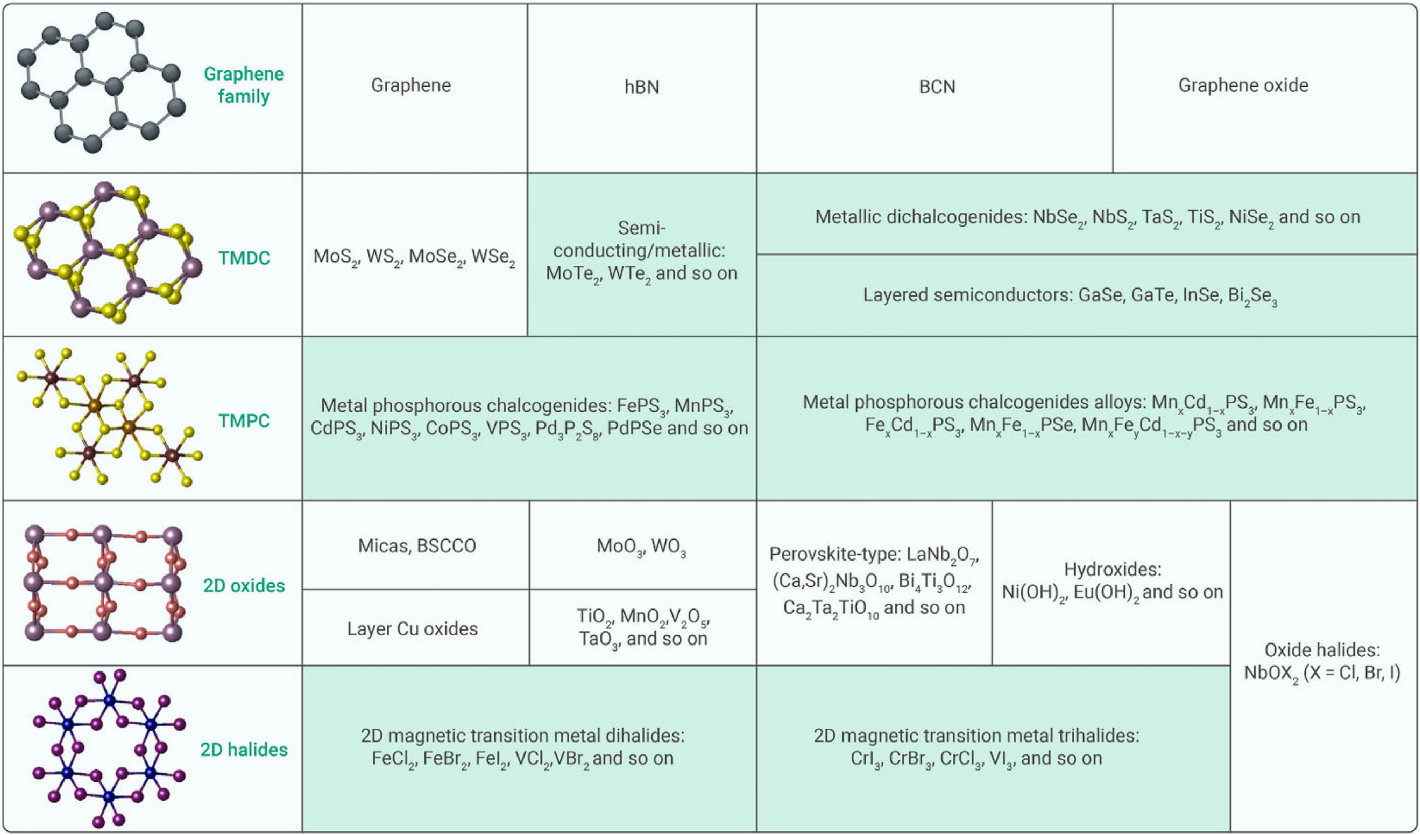
Current 2D library and crystal structures Materials that are unstable in air are shaded in green.^10^^,^^12^

This paper provides a concise overview and presents a snapshot of the current landscape of 2D materials, as illustrated in Figure 1.^9^^,^^10^^,^^11^ The figure demonstrates the remarkable expansion of 2D materials into a diverse and extensive family owing to continuous scientific exploration. Notably, 2D materials have expanded to include metals, semiconductors, and insulators. Compared with traditional 3D materials, 2D materials provide a platform for creating heterostructures with various characteristics. Despite the burgeoning diversity, it is challenging that, although unique physical properties have been discovered from 2D materials such as TMDCs (majority of) and 2D halides,^12^^,^^13^^,^^14^^,^^15^^,^^16^^,^^17^ these materials, especially in the monolayer limit, exhibit an unprecedented sensitivity to oxygen and water.^18^^,^^19^^,^^20^ Therefore, this heightened sensitivity renders them incapable of surviving under ambient conditions, significantly impeding further exploration and practical utilization. The delicate nature of these materials underscores the need for controlled environments in research and applications, as well as emphasizing innovative strategies to mitigate their susceptibility to external factors. This challenge, while formidable, leaves an opportunity for considerable advancements in materials science and conceivably unlocks the full potential of these intriguing 2D materials for quantum applications.

## To investigate the intrinsic structure of air-sensitive materials

### Challenges emerged during the process of using a transmission electron microscope

Since the inception of the transmission electron microscope (TEM) by Ruska in 1932,^21^ electron microscopy has become a transformative force in materials science, physics, and life sciences.^22^^,^^23^ With the continuous development of imaging technology, Pennycook and co-workers proposed the high-angle annular dark-field (HAADF) imaging technique for scanning transmission electron microscopy (STEM),^24^^,^^25^^,^^26^ contributing to the remarkably reinforced contrast between different atomic columns in the sample. STEM scans a sample with a focused incident electron beam. The signal intensity in the HAADF image is nearly proportional to Z^1.7^, where Z denotes the atomic number of the elements in the specimen.^27^^,^^28^^,^^29^^,^^30^ This contrast allows for visualizing different atomic columns in the specimen based on their atomic numbers. Z-contrast imaging is particularly valuable in revealing fine structural details and compositional variations at the atomic scale, enabling it to be a powerful tool for characterizing materials in various scientific disciplines. Electron microscopy’s foundational theory and experimental techniques have experienced rapid evolution since the 1960s. In recent years, aberration correctors and electron monochromators have been widely applied, propelling transmission electron microscopy (TEM) into the sub-angstrom realm. The prevalence of aberration-corrected electron microscopes is poised to exert profound and unpredictable impacts on electron microscopy, materials science, and physics, offering solutions to pivotal structural inquiries while ushering in a new era of challenging research endeavors.

Compared with 3D materials, 2D materials can be obtained directly as thin-layer samples through chemical synthesis or mechanical exfoliation. Therefore, 2D materials require less sample preparation and are more suitable to be characterized for their atomic structure with TEM. Currently, TEM is extensively utilized for the structural characterization and elemental analysis of 2D materials. The basic TEM sample preparation flow of 2D materials requires specific steps to transfer the sample on the substrate (silicon wafer, mica, sapphire, etc.) to the TEM grid.^31^^,^^32^ The transfer steps of this method are described as follows. First, a layer of a polymer such as (PC/PPC/PMMA) is spin coated onto the sample (on a wafer or other substrates).^33^^,^^34^ Then, the polymer film (with sample) is separated from the wafer by HF or NaOH to etch the sample. After the sample is fished with a TEM grid, the organic solvent finally dissolves the polymer covering the sample to complete the transfer process.

Despite scientists' efforts to optimize the sample preparation steps, there are still difficulties in the preparation of 2D material samples. Specifically, (1) most few-layer or monolayer materials, such as air-sensitive TMDCs (Figure 1), are highly susceptible to degradation in the presence of moisture and oxygen.^35^^,^^36^^,^^37^ Thus, they cannot survive during the sample preparation process because of a long time of exposure to ambient conditions. Nevertheless, preparing 2D samples is a time-consuming process that requires sophisticated techniques and expertise. (2) Once the fresh surface of the obtained 2D material samples is exposed to the atmosphere, organic contamination will gradually accumulate on the surface. Consequently, the image quality during TEM imaging is negatively influenced because of the typically few nanometers thickness of 2D material samples.

By fabricating graphite-encapsulated heterostructure, the sample can be prevented from deterioration and beam irradiation.^38^^,^^39^^,^^40^^,^^41^ However, this method only applies to small-area samples dissociated on silicon wafers. Moreover, this method demands high technical expertise from the experimenter and is time-consuming. In contrast, introducing the inert gas atmosphere provided by the glovebox can effectively weaken structural damage and protect the samples during the transfer process. Furthermore, the sensitivity problem of 2D materials can be fundamentally resolved if the whole experimental streamline is protected by an inert gas atmosphere.

### Solution: Inert gas protection during the whole experimental streamline

A glovebox is a sealed container designed to manipulate objects where a separate atmosphere is desired. With its airtight construction and use of inert gases such as nitrogen or argon, the glovebox prevents contamination and preserves the integrity of reactive substances. Burch et al. proposed the "cleanroom-in-a-glovebox" system^42^ as the research on the novel physical properties of water- and oxygen-sensitive materials progressed. This system integrates micro-nano device fabrication techniques by constructing an interconnect glovebox system, enabling the processing of transport devices from sensitive materials. In the pursuit of preparing high-quality TEM samples, nonetheless, intricate tasks and precise sample handling are required during this process. Hence, the environmental interactions and new modifications to the current glovebox system setting should be specially considered to achieve complete water and oxygen isolation from sample synthesis to TEM characterization.

**Figure 2:**
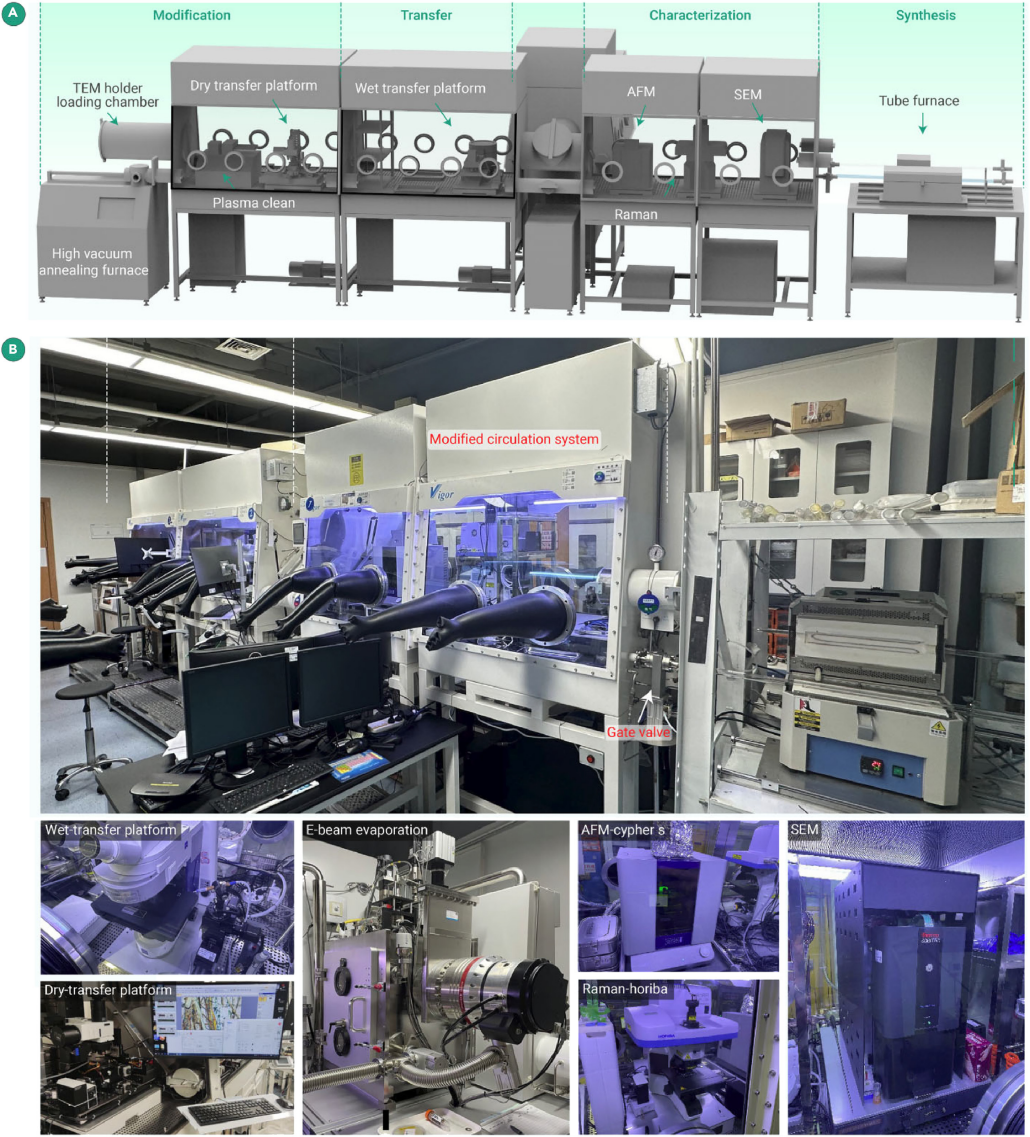
Inert gas-protected GIS for air-sensitive 2D materials (A) Schematic displaying the GIS, the detailed photos for each unit are shown in (B) The purple dashed lines are used to divide different units of the GIS.

To this end, Lin et al. designed a fundamental solution by constructing an inert gas glovebox integrated system (GIS) for the entire experimental process. Using this system, researchers achieved atomic-resolved STEM characterization of water- and oxygen-sensitive samples. The schematic diagram of the GIS is displayed in Figure 2A, and detailed photos of the integrated experimental equipment are presented in Figure 2B. The GIS comprises four main components: modification, transfer, characterization, and synthesis zone.

Each component is housed in a separate glovebox, with average dimensions of approximately 1,800 cm in length, 1,200 cm in width, and 2,500 cm in height. Each glovebox has a custom nitrogen-gas circulation and filtration system to ensure high water and oxygen isolation. The gloveboxes are connected via transition chambers, allowing for sample transfer within an inert gas environment. These units are seamlessly integrated into the GIS to provide efficient operation and precise environmental control for sensitive experiments.

Instruments integrated include atomic force microscope (AFM) (Cypher, Oxford Instruments), scanning electron microscope (SEM) (Phenom Pro), Raman spectroscopy (HORIBA XploRA ONE), fully automated wet and dry transfer platforms and optical microscope (Zeiss), plasma cleaner, and high-vacuum annealing furnace within these units (in Figure 2B). Six chemical vapor deposition (CVD) tube furnaces are connected to the gloveboxes via gate valves. After the material is synthesized, the gate valves can be opened, allowing for the transfer of samples directly from the tube furnaces to the gloveboxes for subsequent processing. In this system, nitrogen is mainly adopted as the inert gas source for the following reasons. Firstly, the physical properties of nitrogen render it suitable for applications in large, streamlined, integrated systems. Since it is lighter than argon and carbon dioxide, it can circulate more quickly and uniformly, so as to ensure consistent environmental conditions. Secondly, nitrogen has a higher ionization threshold than argon and is thus more effective in protecting the electron gun in SEM and other high-voltage instruments because argon can easily be ionized in high voltage. Lastly, nitrogen is cost-effective compared with helium or argon, making it more economical for the continuous supply required in gloveboxes.

Compared with conventional gloveboxes that are able to perform only basic operations such as sample storage or operation, the GIS is the first attempt to unify TEM with an inert gas environment in an integrated system and differs significantly in the following technical details.

(1) Since GIS integrates high-precision experimental instruments such as AFM and SEM, our research group redesigned the system to minimize vibration. The primary vibration sources involve the gas circulation filtration system and the connected vacuum pump. Under this consideration, the circulation system was separated from the main glovebox chamber. In addition, each instrument was placed on an active vibration isolation table, and each vacuum pump was placed on a shock-absorbing layer to prevent vibrations from being transmitted to the ground.
(2) For the purpose of avoiding the accumulation of static charge that commonly occurs within the glovebox system, large static meters and static eliminators were installed in each glovebox to minimize static generated by glove friction. Each precision instrument was equipped with a shielding layer to mitigate interference from static on the instruments. Besides, a dedicated ground line was introduced for electrical transport devices to dissipate excess charge.
(3) The GIS was incorporated with several instruments and motors (transfer platform), especially the electron gun in the SEM, which generated substantial heat. The glovebox required efficient cooling to prevent high temperatures from damaging the instruments. Thus, a circulating cooling water system connecting all the gloveboxes was installed to maintain the temperature around 25°C.
(4) Since the wet transfer process introduced additional water and organic solvents into the glovebox, this section of the glovebox’s circulation system was specially designed to better adsorb these vapors. The frequency of filter replacement and circulation regeneration was increased to maintain the removal capability of these components.
(5) All transfer platforms were designed to be fully automatic, allowing researchers to perform transfer operations outside the box, dramatically lessening workload, and effectively strengthening transfer accuracy.
(6) A large transfer chamber was incorporated to accommodate the larger vacuum TEM holder. In this way, the sample can be loaded onto the vacuum TEM holder within the glovebox before being transferred to the microscope.

The workflow of this system is briefly introduced as follows. Firstly, our research group fabricated sensitive 2D samples through a tubular furnace using CVD that was directly connected to the glovebox or applying mechanical exfoliation from bulk materials inside the inert gas environment. The fresh, sensitive 2D samples were analyzed for their elemental composition, thickness, and phase identification by the built-in AFM, SEM, and Raman spectroscope, as illustrated in Figure 2B. Then, the sample was transferred within the GIS for TEM sample, with an innovative approach that effectively improved the transfer precision for 2D material by the wet transfer. Specifically, a semi-automatic wet transfer stage was designed to compose a vacuum-adhesive needle incorporated with a three-axis electrically controlled robotic arm, enabling micrometer-level precise positioning control of TEM grids. Under optical microscopy observation, the sample was aligned precisely with the designated position on the TEM grid. Subsequently, the grid adhered to the silicon wafer by releasing the vacuum, and the sample was precisely transferred onto the TEM grid through HF etching. This GIS also incorporated a fully automatic dry transfer platform and thus can support meticulous encapsulation through graphene or boron nitride to fabricate 2D heterostructures. This ensures the protection of e-beam-sensitive samples and facilitates the preparation of devices for transport measurements.

After the sample preparation, our research group removed the organic residue using ultra-high vacuum annealing connected to GIS to minimize the sample contamination. In addition, a home-built semi-automatic wire bonding instrument with a self-design sealing puck was integrated inside the GIS for the final wiring of the device to electrodes. Ultimately, the as-fabricated sample (TEM grid or device on wafers) was characterized by various homemade portable vacuum holders placed inside GIS. With the intention of measuring their intrinsic physical properties, these holders were designed to connect to diversified advanced instruments such as physical property measurement systems (PPMSs) and scanning tunneling microscopy (STM). Moreover, the pristine structure and physical properties of air-sensitive 2D materials were preserved and measured by introducing the novel GIS.

**Figure 3:**
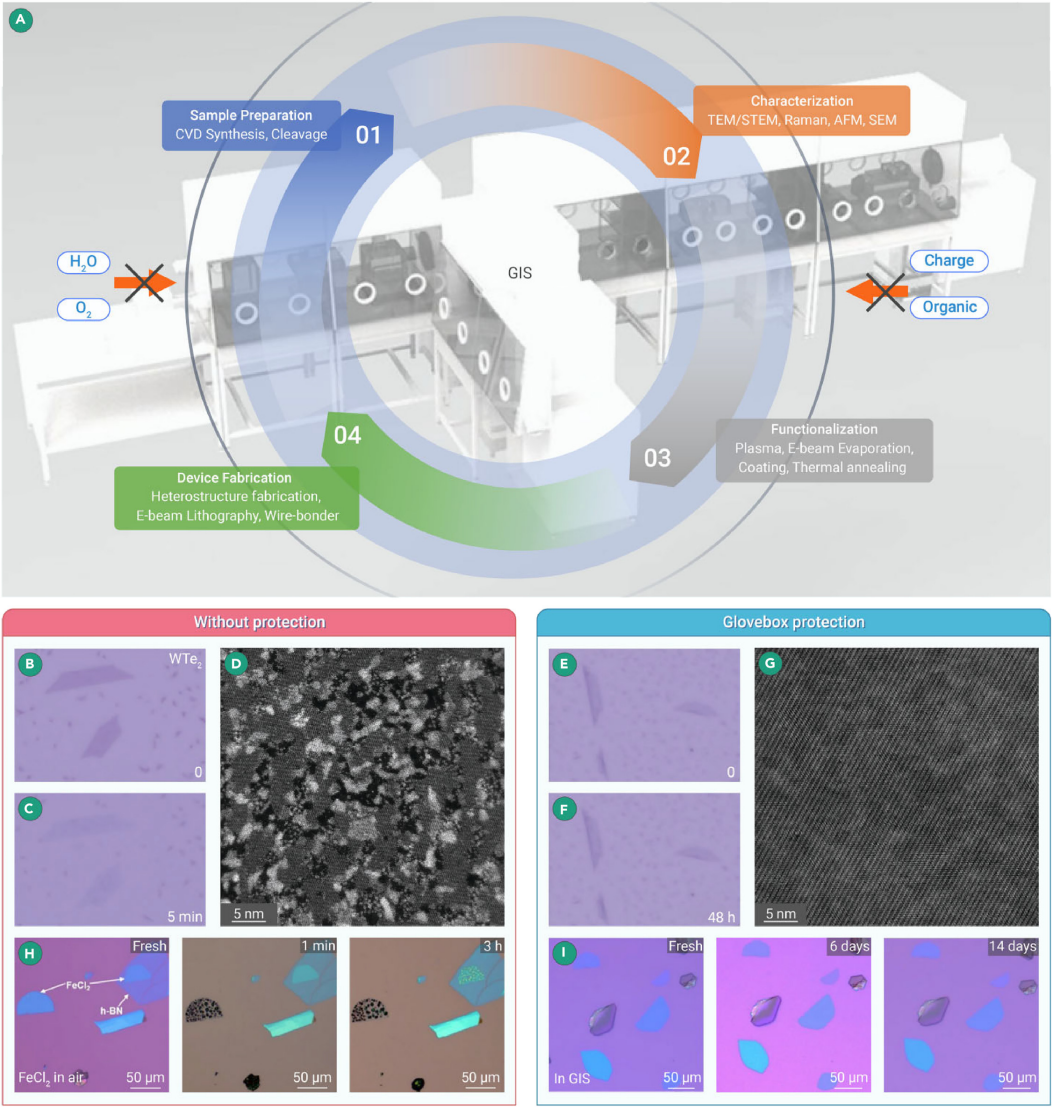
GIS for air-sensitive 2D materials (A) (i and ii). Schematics of GIS. The GIS enables the full isolation of water and oxygen through the whole process. Optical images of 1T′-WTe_2_ exposed in air (B) right after growth and (C) after 5 min; optical images of 1T′-WTe_2_ in the glovebox (E) right after growth and (F) after 48 h. Large-scale high-angle annular dark-field (HAADF) STEM images of large-scale WTe_2_ monolayer prepared (D) in air and (G) in the glovebox, respectively.^44^ (H) Optical images of h-BN encapsulated fresh FeCl_2_ flakes, and after 1 min and 3 h under ambient conditions. Note that the flakes without any protection deliquesced immediately upon exposure to air. (I) Optical images of FeCl_2_ fresh flakes placed in the glovebox after 6 and 14 days.^15^

In summary, this system comprises wholly isolated water and oxygen during the sample synthesis (using bottom-up methods such as CVD),^43^ sample functionalization through multiple approaches, device fabrication, and transport property measurements to structural characterization of sensitive 2D materials, as exhibited in Figure 3A. To ascertain the critical role of inert gas protection in the characterization of water-sensitive 2D materials, Niu et al. systematically compared the basal lattice of CVD-synthesized monolayer WTe_2_ prepared both in air and GIS (Figures 3B–3G), thereby confirming the detrimental effects of oxygen on the material.^44^ As mentioned, the surface structure is easily oxidized and degraded in minutes owing to the metastability of the air-sensitive 1T′-WTe_2_ monolayer. When sensitive 2D materials are exposed to the atmospheric environment, oxidation reactions lead to a noticeable change in the contrast and color of the materials under optical microscopy observation.^45^^,^^46^ In their experiments, the 1T′-WTe_2_ monolayer was exposed to air for 5 min, as demonstrated in Figures 3B and 3C, in which a significant contrast reduction was observed. STEM characterization of the exposed sample revealed that the intense oxidation destroyed the sensitive WTe_2_ monolayer, generating numerous oxidized nanoparticles and contaminants that disrupted the lattice’s periodicity. However, optical microscopy images revealed no significant change in contrast when the 1T′-WTe_2_ monolayer was left in an inert environment within a glovebox for 48 h (Figures 3E and 3F). As a control, subsequent sample preparation processes were all conducted within the GIS. Figure 3F suggests that the large-scale intrinsic lattice structure of monolayer WTe_2_ was preserved.

Another class of more sensitive 2D materials is transition metal dihalides (TMDHs) or trihalides (TMTHs).^47^^,^^48^^,^^49^^,^^50^^,^^51^^,^^52^ When exposed to the atmospheric environment, TMDHs, especially chloride (Cl) compounds, will rapidly adsorb moisture and undergo deliquescence. Therefore, apart from maintaining an inert gas atmosphere in a glovebox for protection, no liquids should be introduced during the sample transfer process to characterize TMDHs by a TEM. Jiang et al. reported the growth of 2D TMDHs in the GIS.^15^ The as-grown FeCl_2_ flake was placed in the atmosphere and glovebox for comparison to unveil the sensitivity of TMDHs FeCl_2_. The optical images are presented in Figures 3H and 3I. Notably, apparent degradation was observed within 3 h, even though h-BN encapsulated the FeCl_2_ flakes, presenting the extreme sensitivity of the TMDHs. However, negligible change in the sample was confirmed when the flake was placed in the glovebox system for 14 days.

The entire sample preparation process must be conducted within an inert gas atmosphere-protected glovebox to successfully characterize sensitive 2D materials. Besides the glovebox system, the vacuum system also plays a vital role in preparing sensitive 2D material samples. Samples prepared within vacuum systems exhibited high quality with minimal contamination. Simultaneously, the ultra-high-vacuum environment can effectively avoid the formation of bubbles at the interface during the fabrication of 2D heterostructure, rendering them suitable for device transfer tests and high-quality 2D heterostructure fabrication.^53^^,^^54^ Nevertheless, the nature of the vacuum system is nearly incompatible with TEM, including sample fabrication and loading, because the sample must be free-standing for transmission operation. For TEM sample preparation, researchers must perform intricate tasks requiring precise sample handling and more extensive operation space. In addition, it is challenging to achieve in a vacuum system considering that performing wet transfers involves the introduction of solvents. In such cases, glovebox systems are more suitable due to their relatively low cost, ability to accommodate large volumes, and compatibility with solvents. Unfortunately, the limitations of system size, precise sample handling, and the introduction of solvents significantly constrain the scope and scale of TEM sample preparation experiments that can be conducted within a vacuum system. A more feasible and cost-effective way is to develop a vacuum transfer system into the GIS to connect to any other vacuum system.

The experimental results also demonstrate enhanced resistance to beam damage of the sample prepared in GIS. Apart from preventing sample oxidation and deliquescence, our experimental observations indicate that the rate of electron beam damage was indeed influenced by the level of organic contamination of the samples, which commonly stemmed from hydrocarbon contaminants during the process of preparing samples in air. Given that the electron beam damage to samples is directly correlated to the knockon damage (accelerating voltage) and radiolysis damage (beam current of the electron microscope), our research group first assumed that the damage tolerance of knockon and radiolysis should be nearly the same for the samples prepared in air and within the GIS. However, there is another damage mechanism named beam-assisted chemical etching. During imaging, organic contaminants tend to cause charge accumulation and thus beam-assisted chemical etching that is more rapid in heavily contaminated samples. For instance, clean monolayer WTe_2_ seems more robust to the electron beam than the heavily contaminated one from exposure to a high organic vapor concentration. Therefore, the clean sample prepared in GIS can somewhat strengthen resistance to beam damage.

## Results from the GIS and other glovebox systems

### Sensitive 2D TMDCs

2D TMDCs have attracted considerable interest in fundamental research due to their unique physical properties, such as direct band gaps, strong spin-orbit coupling, and excellent electronic and mechanical performance.^55^^,^^56^^,^^57^ Currently, 2D TMDCs have been applied to spintronics,^58^ optoelectronics,^59^ energy harvesting materials,^60^^,^^61^ and flexible electronics.^62^ MoTe_2_ and WTe_2_,^63^^,^^64^^,^^65^^,^^66^ as representative 2D materials in the TMDCs family, have also become research hot spots. Each layer of MoTe_2_ (WTe_2_) consists of alternating arrangements of Mo (or W) and Te atoms, forming a 2D layered structure. 2D TMDCs materials have rich phase structures, and the three typical phases are characterized by either trigonal prismatic (2H), octahedral (1T), or dimerized (1T′) phases.^67^^,^^68^^,^^69^^,^^70^ The difference between these phases involves changing the layer’s stacking order (2H ABA and 1T ABC) or different bonding types (metal-metal bond formed in 1T′). In addition, the relatively small energy gap between phases enables phase engineering. The layered structure of MoTe_2_ and WTe_2_ has endowed them with many unique electronic properties. With the introduction of external stimulations, such as applying an electric field or strain, WTe_2_ and MoTe_2_ exhibit peculiar physical phenomena, comprising tunable band structures, topological properties,^71^ and strong correlation effects. These properties make MoTe_2_ and WTe_2_ highly promising for designing novel devices and achieving functional applications.

WTe_2_ and MoTe_2_ are prominent TMDCs that have attracted considerable attention due to their unique topological and quantum properties. In specific crystal phases, such as the 1T′ phase, both materials demonstrate type II Weyl semimetal characteristics,^72^^,^^73^ where the conduction and valence bands tilt and intersect, forming Weyl points. Topologically protected surface states appear on these Weyl points, leading to distinctive quantum transport phenomena.

In addition, WTe_2_ and MoTe_2_ exhibit notable anisotropic electrical conductivity and giant magnetoresistance at low temperatures.^65^^,^^74^^,^^75^^,^^76^ Specifically, a significant increase in resistance was observed in WTe_2_ under an applied magnetic field, making it ideal for investigating quantum magnetoresistance effects. Both materials have also demonstrated superconducting behavior under extreme conditions, such as low temperatures or high pressure,^77^^,^^78^^,^^79^ prompting interest in their potential as topological superconductors.

WTe_2_ and MoTe_2_, as layered materials, can be prepared in monolayer or few-layer structures through mechanical exfoliation or CVD,^70^^,^^80^ rendering them highly attractive for use in 2D device fabrication. Their unique properties and ease of fabrication establish WTe_2_ and MoTe_2_ as essential members of the family of 2D topological and quantum materials, driving the development of novel quantum devices. All of these studies depended on the quality of samples considering that the early-stage preparation process of sensitive 2D materials directly impacts their performance.

However, few-layer MoTe_2_ and WTe_2_ exhibit sensitivity to water and oxygen, making them unstable under ambient conditions. Consequently, it is challenging to observe their large-scale and intrinsic atomic structure directly. With the purpose of preparing 2D TMDCs samples for TEM characterization or device transport under ambient conditions, the sample degradation can be effectively prevented by fabricating graphite/h-BN encapsulated heterostructures.^81^^,^^82^^,^^83^ The drawback of this method is extra transfer, which is time-consuming and not applicable to CVD-grown samples.

**Figure 4:**
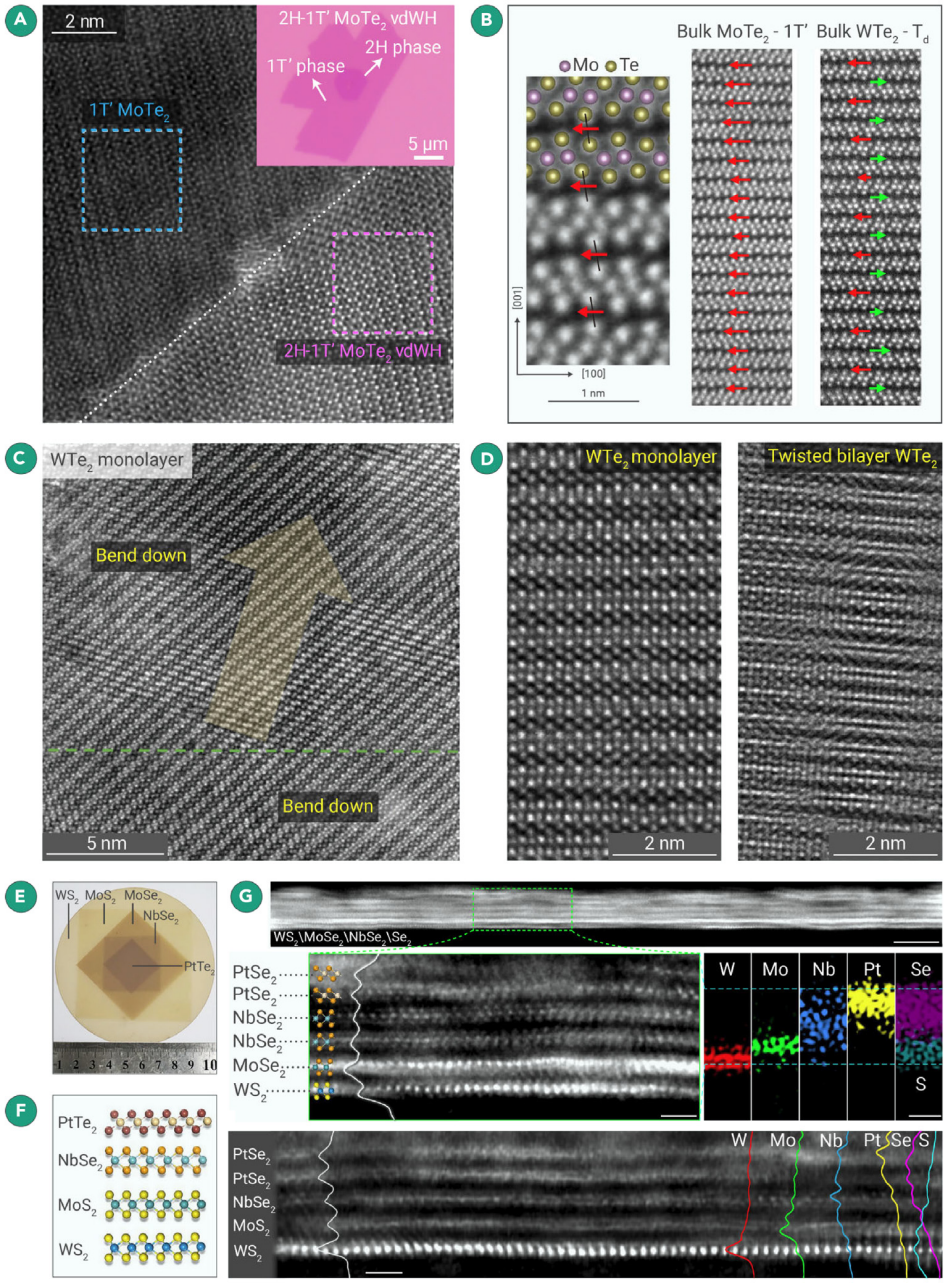
STEM characterization of 2D TMDCs under glovebox protection (A) Atomic-resolution image of the step edge in the bilayer MoTe_2_ vdWHs, showing clear 1D zigzag chains of the 1T′ and the hexagonal pattern of the 2H phase, respectively. The upper right corner is the optical image of 2H-1T' MoTe_2_ vdWHs.^70^ (B) Cross-section STEM of bulk 1T′ MoTe_2_ and Td WTe_2_. Black lines indicate spanned Te-Te pairs, and arrows indicate the average interlayer displacement.^84^ (C) Atomic-resolution image of the step edge in the bilayer MoTe_2_ vdWHs, showing clear 1D zigzag chains of the 1T′ and the hexagonal pattern of the 2H phase, respectively.^44^ (D) The STEM images of monolayer WTe_2_ and bilayer twisted WTe_2_.^85^ (E and F) (E) Optical microscope of the as-grown multilayer superconducting heterojunction image. The stacking schematic of WS_2_/MoS_2_/NbSe_2_/PtTe_2_ is shown in (F). (G) Cross-sectional STEM image and EDS mapping of multilayer superconducting heterojunction.^86^

The introduction of the GIS offers researchers a platform with fast and clean sample preparation under inert gas protection. For instance, Guo et al.^70^ utilized a one-step growth method to fabricate controlled bilayer 2H-1T′ MoTe_2_ van der Waals heterostructures in the GIS (as shown in Figure 4A). The tube furnace used for synthesis is directly connected to GIS, followed by wet transfer and vacuum transfer using a vacuum holder for STEM characterizations, so as to avoid oxidation and contamination. Atomic-resolution HAADF-STEM images demonstrate the structural features of uncontaminated large-scale bilayer heterostructures and the distinct moiré patterns. Furthermore, angle-resolved polarized Raman spectroscopy and first-principles calculations suggest that the stacking of the epitaxial bilayer vdW heterostructures modulates interlayer coupling through resonant vibrational modes. Using a similar protection strategy, Hart et al. discovered a highly disordered stacking structure in MoTe_2_ thin flakes (Figure 4B) by the confined energy along the c axis,^84^ and it differs from bulk materials. This finding is crucial for understanding the peculiar physical phenomena in MoTe_2_ thin flakes and implies that c axis confinement may affect the layered stacking of other 2D materials.

Moreover, Niu et al. utilized a similar approach to directly visualize the large-scale, intact lattice structure of air-sensitive monolayer 1T′-WTe_2_^44^ (Figure 4C). They discovered the presence of anisotropic lattice undulations within the monolayer 1T′-WTe_2_ material, which propagates perpendicular to specific lattice planes in the monolayer. This anisotropic lattice undulation provokes intrinsic defects (tellurium vacancies) to aggregate on the inner side of the undulating structure owing to asymmetric strains induced by undulations. Yuan et al. reported that high-quality single-layer and twisted bilayer WTe_2_ samples were fabricated and characterized through mechanical exfoliation and the fabrication of a graphite encapsulated heterostructure by a 2D transfer platform in an inert-gas-protected glovebox (Figure 4D).^85^ They observed that twisted bilayer WTe_2_ with twist angles of approximately 5° and 2° preserved its original moiré structure. The achievement of atomic-resolution visualization for monolayer and twisted bilayer WTe_2_ allows us to understand the material’s microstructure and properties better, laying a foundation for future research and applications involving these materials.

Zhou et al. achieved controlled growth of wafer-scale multiblock vdW superconductor heterostructures^86^ (Figures 4E–4G). The pristine multilayer structure and the unique physical properties of the air-sensitive superconducting heterostructure thin films can be preserved under inert gas protection provided by the glovebox system. In Figure 4G, atomic-resolution cross-sectional STEM images reflect that all the 2D materials in the vdWSHs were intact with a clean vdW gap. This stacking growth method enabled the fabrication of centimeter-scale proximity-induced superconductivity and superconducting Josephson junctions.

**Figure 5:**
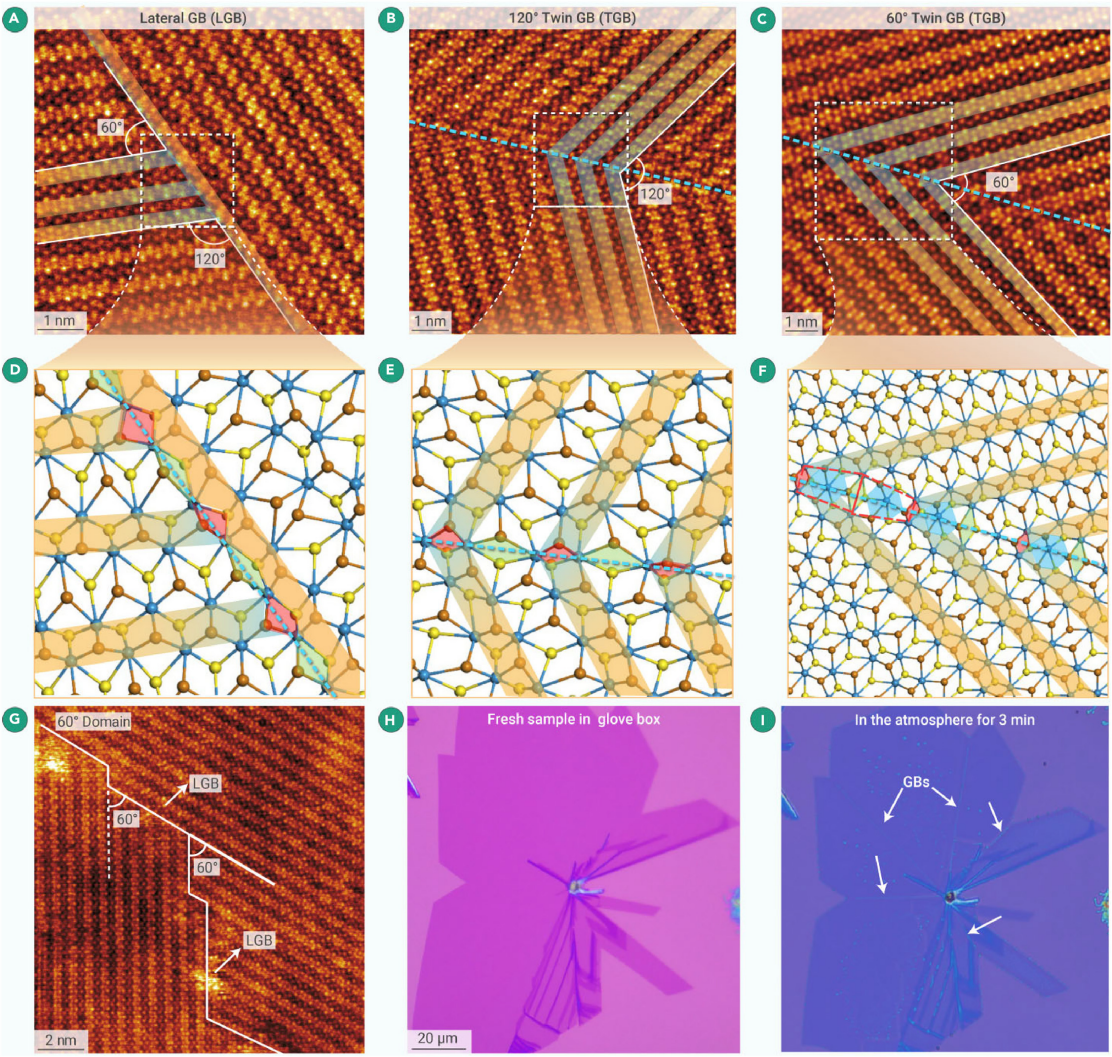
Atomic structure of the GBs in monolayer WTe_2_ (A–C) Corresponding atom-scale HAADF-STEM images of LGB (A), 120° twin grain boundary (TGB) (B), and 60° TGB (C). The quasi 1D W-Te atom chains, featuring short W-Te bonds, which are a crucial structural characteristic of the distorted 1T′ phase, are visually emphasized by yellow ribbons along the [100] direction. (D–F) Related atomic models based on the dotted squares in the STEM images. The W-Te rhombus with saturated coordination (6-fold for W and 3-fold for Te) along the GBs are shaded in green and red colors (D and E), respectively. Additional 6-fold coordinated W atoms between anisotropic vertical W-Te rhombi are shaded in blue in (F). (G) Typical cases of continuous kinked GB pathway at the atomic scale, which extends at certain fixed deflection angles depending on the alternation of the three GB prototypes without generating dislocations in 60° misoriented grains. (H) Optical micrographs of a large area of freshly grown single-layer WTe_2_ film in the glovebox. (I) Optical micrographs of a large area of WTe_2_ film exposed to air for 3 min.^80^

The grain boundary (GB) structure of TMDCs materials demonstrates numerous novel physical properties. Because of these structures' high reactivity nature, however, it is challenging to preserve their intrinsic properties in atmospheric conditions. Consequently, previous studies have not presented effective methods for observing these structures. To overcome this challenge, the GIS offers an excellent solution. Using GIS, Guo et al. revealed the characterization of intrinsic GB structures of air-sensitive monolayer 1T' WTe_2_ using the nucleation-controlled CVD method.^80^ As illustrated in Figures 5A–5F, they identified three primary GB structures in bicrystalline WTe_2_ sheets formed by the stitching of anisotropic edges. These three GB structures served as fundamental units for constructing various GB kinks and influenced the specific arrangement of GBs in continuous monolayer films (Figure 5G). STM/spectroscopy confirmed the presence of enhanced 1D metallic states along the 120° twin grain boundary. Notably, rapid oxidation and degradation occurred at the highly active GB regions once the synthesized sheets were placed in the air for about 3 min (Figures 5H and 5I). These studies validate that the GIS excels in characterizing the intrinsic structure of sensitive materials. Its protective environment allows researchers to unveil previously unobservable structures and thereby elucidate the origins of numerous novel physical properties.

In short, the GIS has successfully addressed the significant challenge of working with few-layer and monolayer MoTe_2_ and WTe_2_, which are highly sensitive to water and oxygen. Moreover, this system can lessen the workload for researchers by eliminating the need for additional encapsulation processes and thus broaden the applications of the air-sensitive TMDCs materials. This achievement has laid a solid foundation for further research on sensitive 2D materials.

### 2D magnetic materials

In 2D materials, thermal fluctuations hinder spin coupling for magnetic order formation. Nevertheless, the robust magnetic anisotropy inherent in 2D materials can mitigate these thermal fluctuations, bringing extensive magnetic coupling and creating stable, ordered magnetic structures. Despite the impact of thermal fluctuations, the persistence of a magnetically ordered state in 2D materials is maintained, which is attributed to the prevalence of magnetic solid anisotropy, rendering them potentially applicable in spintronics devices. The structural manipulation of 2D heterostructures is crucial to realize efficient magnetic control and superior performance. TMDCs have garnered considerable attention because of their rich electronic properties and non-bonded layered surface structure and become promising candidates for crafting captivating 2D heterostructures.^87^ Among thousands of TMDC compounds, chromium telluride (Cr_*x*_Te_*y*_) is a prime illustration of intercalated TMDCs, showcasing intriguing intrinsic magnetism.^88^^,^^89^^,^^90^^,^^91^^,^^92^^,^^93^ In the burgeoning field of 2D magnet research, chromium telluride captures significant focus and holds substantial promise as a robust contender for high-temperature 2D ferromagnets. However, certain amorphous oxidation layers appeared on the surface sample after sample preparation under ambient conditions, which is ascribed to the sensitivity when exposed to water and oxygen.^88^^,^^94^ Therefore, the indispensable use of a glovebox for protection should be emphasized to perform structural characterization of these materials.

**Figure 6:**
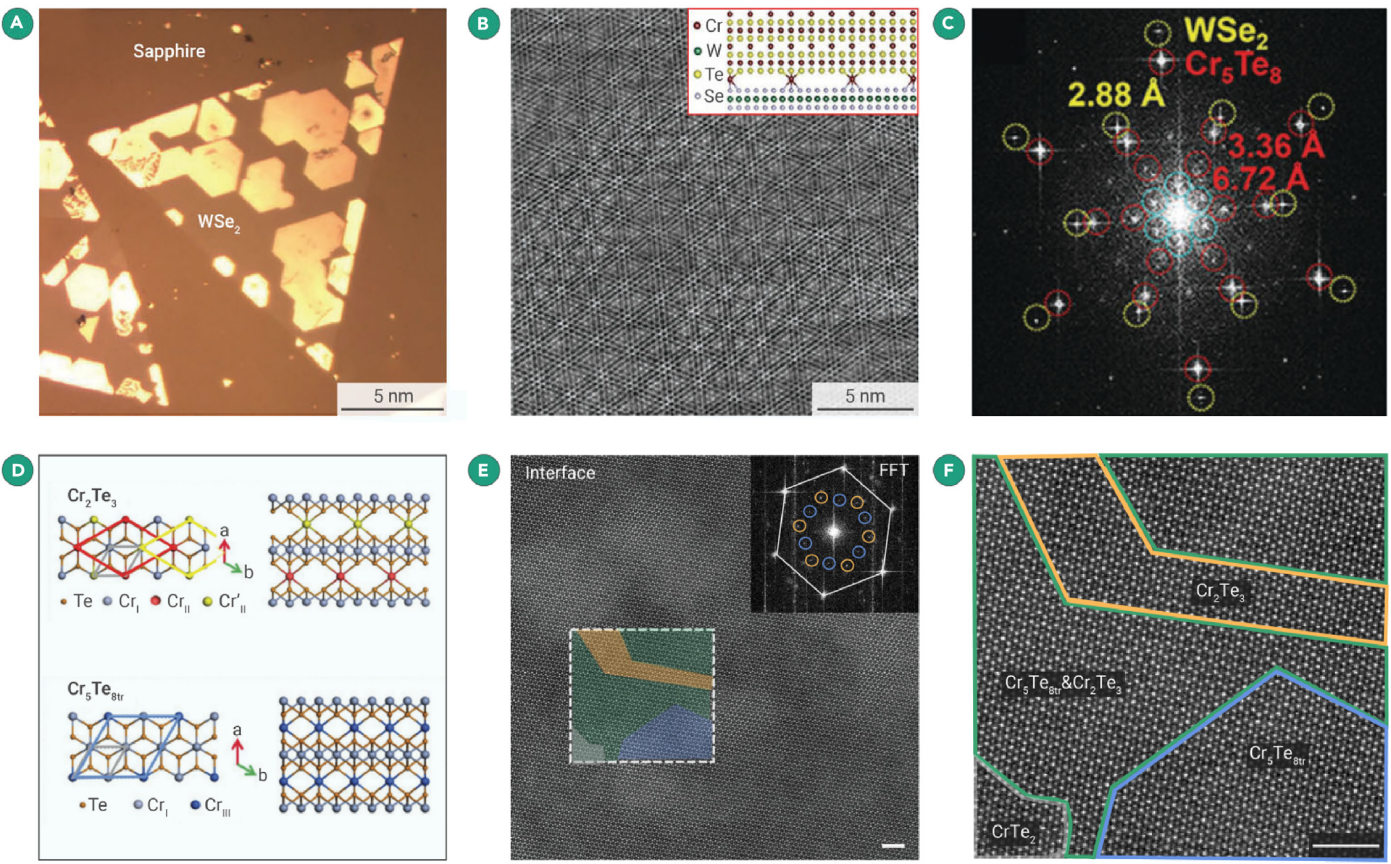
2D magnetic materials (A) 2D heterostructure of Cr_5_Te_8_/WSe_2_. (B) HAADF-STEM image showing the Moiré pattern of the Cr_5_Te_8_/WSe_2_ heterostructure. The inset depicts a top view of the Cr_5_Te_8_/WSe_2_ superlattice atomic model along (210).^96^ (C) FFT image of (B) with diffraction points labeled: WSe_2_ (100) marked by yellow circles, Cr_5_Te_8_ (200) marked by outer red circles, interlayer Cr atoms matching the triangular Cr_5_Te_8_ marked by inner red circles, and Moiré superlattice marked by blue circles.^96^ (D) Crystal structures of Cr_2_Te_3_ and Cr_5_Te_8tr_ along different axes. The gray rhombus represents the first-order periodic CrTe_2_ unit cell with a lattice spacing of 0.34 nm. Red/yellow and blue rhombi represent the superlattice caused by the insertion of Cr_II_/Cr_II'_ and Cr_III_ atoms in Cr_2_Te_3_ and Cr_5_Te_8tr_, respectively.^98^ (E) High-resolution image of the heterojunction interface region. The inset shows the corresponding FFT. The gray hexagons indicate the first-order Bragg peaks contributed by the CrTe_2_ framework. The orange and blue circles represent the fine super-periodicity caused by the insertion of Cr atoms in Cr_2_Te_3_ and Cr_5_Te_8tr_, respectively.^98^ (F) Zoomed-in image of the quadrilateral box in (E).^98^

For instance, Bian et al. reported the 2D chromium telluride CVD growth, with a Curie temperature of 180 K, a large perpendicular anisotropy of 7 × 10^5^ Jm^−3^, and a high coercivity of ∼4.6 kG at 20 K.^95^ Atomic-resolution HAADF-STEM and energy-dispersive X-ray spectroscopy (EDS) analysis identified the intrinsic crystal structure as Cr_2_Te_3_ without deterioration by performing the sample preparation in GIS. DFT calculation further indicates that Cr_2_Te_3_ undergoes canted to collinear ferromagnetism, transiting from perpendicular to in-plane anisotropy with varying thickness from bulk to atomically thin Cr_2_Te_3_. They also achieved the growth of a dative epitaxy 2D magnetic material Cr_5_Te_8_/WSe_2_ vdW moiré superlattice through CVD (Figure 6A).^96^ Subsequently, they transferred and characterized the structure of the samples under the protection of an inert gas atmosphere provided by GIS. A HAADF-STEM image (Figure 6B) reveals a distinctive moiré pattern. Fast Fourier transform (FFT) patterns (Figure 6C) demonstrated three sets of diffraction spots with hexagonal symmetry, marked in yellow, red, and blue circles, respectively. This confirms the epitaxial growth of covalent 2D Cr_5_Te_8_ crystals on monolayer WSe_2_. The magnetic hysteresis loops exhibited nearly perfect square hysteresis, suggesting the absence of domain wall pinning by defects. Moreover, Tang et al. discovered a robust ferromagnetism with strong perpendicular anisotropy in the synthesized trigonal and monoclinic Cr_5_Te_8_.^97^ A high Curie temperature of up to 200 K with an anomalous Hall conductivity of 650 Ω^−1^ cm^−1^ and anomalous Hall angle of 5% can be obtained in distorted monoclinic Cr_5_Te_8_ through phase engineering. Under GIS protection, the intrinsic atomic structure for both phases is clearly resolved through STEM.

In the case of self-intercalation of Cr atoms in 2D Cr_*x*_Te_*y*_, Niu et al. applied a one-step CVD synthesis method and characterized the lateral heterostructure of Cr_2_Te_3_ and triangular Cr_5_Te_8_ (Cr_5_Te_8tr_) in the GIS.^98^ The atomic model of the Cr_2_Te_3_-Cr_5_Te_8tr_ heterostructure is displayed in Figure 6D. Cr_III_ atoms were collectively embedded beneath the Cr_I_ atomic columns with a half-filled rate, forming a (2 × 2) supercell of CrTe_2_. The central and edge CrTe_2_ chains shared the basis of the main chain, while the ordered intercalation of Cr_II/III_ atoms formed new phases of Cr_2_Te_3_ and Cr_5_Te_8tr_ with larger lattice constants. A mixed ordering of the Cr_2_Te_3_ and Cr_5_Te_8tr_ phases was observed in the transition region of the heterostructure. Figure 6E provides the atomic-resolved HAAD-STEM image in a phase boundary region between the Cr_2_Te_3_ and Cr_5_Te_8tr_ phases along the [001] axis. The corresponding FFT pattern reflects the coexistence of two superlattice peaks, marked with different colored circles (orange for Cr_2_Te_3_ and blue for Cr_5_Te_8tr_). The above works unveil the promising applications of Cr_*x*_Te_*y*_ material in future magnetoelectronic and spintronic devices. Notably, the GIS effectively protected the sensitive structure of 2D magnetic Cr_*x*_Te_*y*_, laying a solid foundation for understanding the ferromagnetism properties arising from its intriguing structure-function relationships.

### 2D transition metal halide

Transition metal halide (TMH) compounds are formed by transition metals (such as titanium, chromium, and vanadium) and halogen elements (such as fluorine, chlorine, bromine, and iodine), comprising 2D transition metal dihalides (MX_2_) and trihalides (MX_3_). Some TMH compounds exhibit strong spin-orbit coupling effects, leading to complex magnetic behaviors, involving ferromagnetic, antiferromagnetic, and ferrimagnetic states. The rich magnetic orders in MX compounds can be precisely modulated by stress control, stacking, and other methods that alter interlayer van der Waals forces coupling. This diversity makes TMH compounds ideal candidates for research and applications. Their unique magnetic properties lay a foundation for novel storage devices and provide more options for applications such as magnetic sensors.

**Figure 7:**
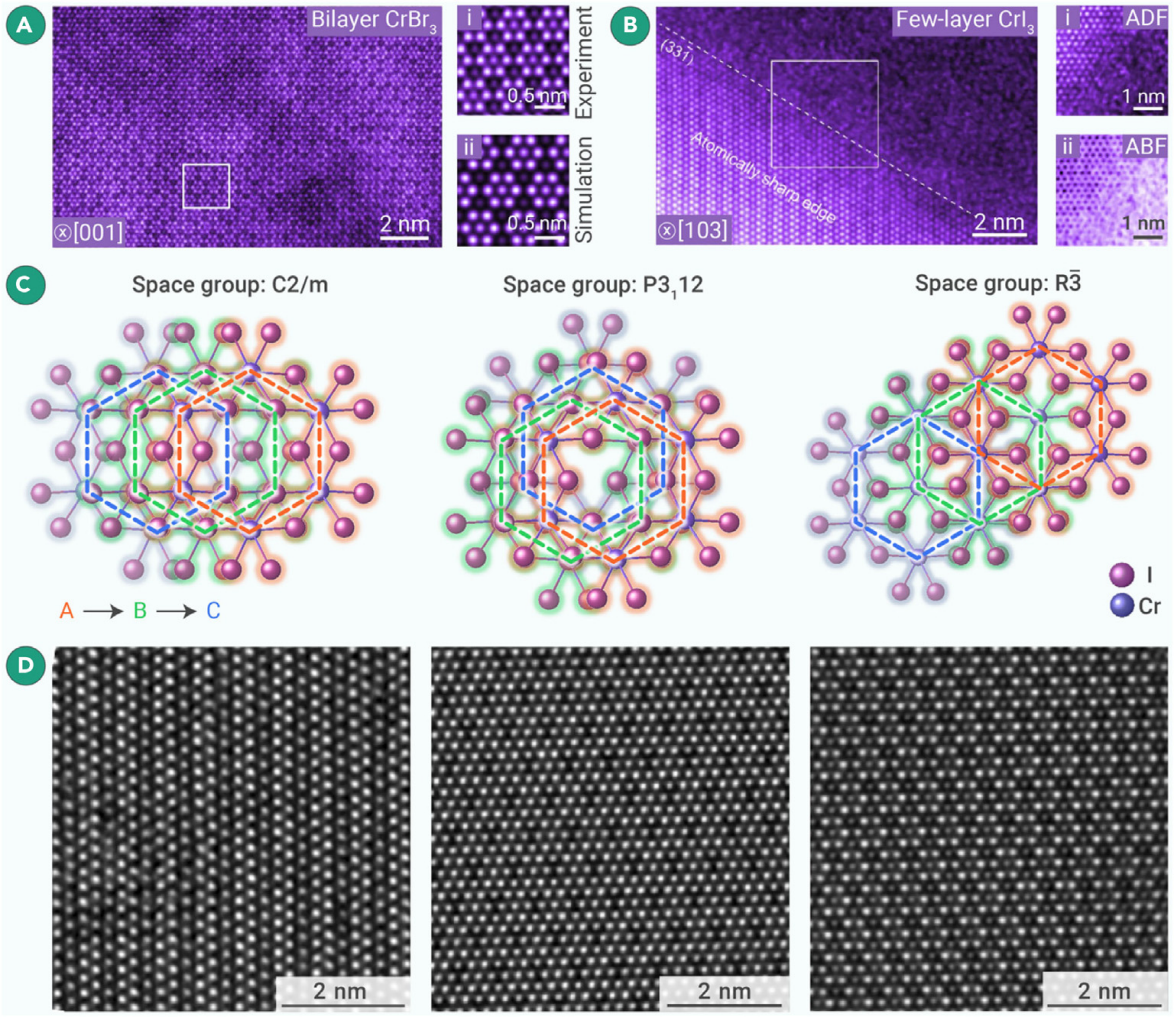
STEM characterization of 2D transition metal trihalides Atomic-resolution STEM images demonstrating the success of the dry transfer method of (A) dual-layer CrBr_3_ with the experimental and simulated image shown in (A(i) and A(ii)). (B) Atomic-level thin layer of CrI_3_, with the ADF imaging and annular bright-field (ABF) imaging shown in (B(i) and B(ii)).^99^ (C) Atomic structure models of different stacking types of CrI_3_ observed along the c axis in the C2/*m*, P3_1_12, and *R*-3 phases. (D) Atomic-resolution ADF-STEM images corresponding to (C) from left to right.

Most TMH compounds are highly susceptible to moisture and oxygen in the air. Therefore, it is crucial to employ inert gas protection and isolate water and oxygen during the material synthesis and transfer processes. Han et al. utilized a glovebox system to perform the dry transfer of TMH compounds and realized the characterization of different stacking configurations of atomically thin MX_3_ crystals.^99^ Figure 7A presents an STEM image of bilayer CrBr_3_ successfully transferred by the dry transfer method, fitting well with simulated images. This method is also highly effective for air-sensitive CrI_3_, enabling atomic-thin CrI_3_ to maintain a good structure in the edge region without significant degradation (Figure 7B).

To better reveal the CrI_3_ stacking structures, Hou et al. applied graphite encapsulation to the few layers of CrI_3_ using the GIS. Figure 7C illustrates several stacking types of CrI_3_: (1) C_2_/*m* monoclinic stacking, (2) *P*3_1_12 triangular stacking, and (3) *R-*3 rhombohedral stacking, with corresponding ADF-STEM images along the c axis (Figure 7D). These works unveil the potential mechanism of achieving engineered magnetic heterostructures and textures through interlayer van der Waals coupling within the same material.

**Figure 8:**
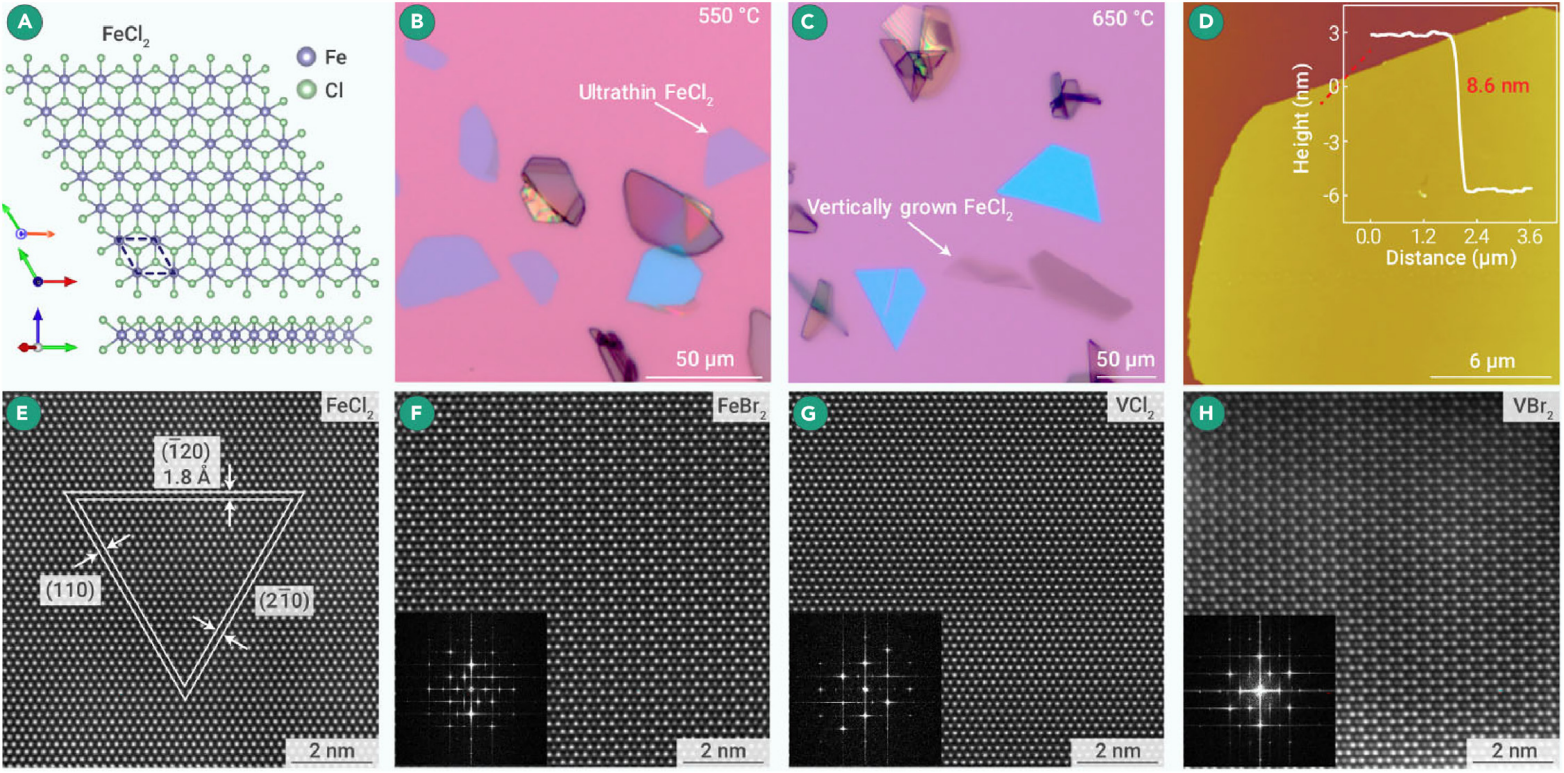
STEM characterization of 2D transition metal dihalides (A) Top and side views of the 1T-FeCl_2_ crystal structure. Purple represents Fe, and green represents Cl.^15^ (B) Optical microscope (OM) image of the FeCl_2_ thin film synthesized at 550°C. (C) OM image of the FeCl_2_ thin film synthesized at 650°C. (D) AFM image of the FeCl_2_ thin film and corresponding height distribution. (E) Atomic-scale HAADF-STEM image of the FeCl_2_ thin layer.^15^ (F) HAADF-STEM image of the thin layer of VCl_2_, with the inset showing its corresponding FFT pattern.^15^ (G) HAADF-STEM image of the thin layer of VBr_2_, with the inset showing its corresponding FFT pattern. (H) HAADF-STEM image of the thin layer of FeBr_2_, with the inset showing its corresponding FFT pattern.

Moreover, the preparation of MX_2_ can also be achieved through the reduction of MX_3_, as reported by Jiang et al., who prepared high-quality 2D magnetic transition metal dihalides using a home-built glovebox system and the halide reduction method and provided a universal approach for MX_2_ synthesis (1T-FeCl_2_, FeBr_2_, VCl_2_, and VBr_2_).^15^ In their work, FeCl_2_ flakes grown on SiO_2_/Si were either laid down or vertically grown on the substrates. In this case, the TEM specimen was easily prepared by directly pressing the vertically grown FeCl_2_ flakes on SiO_2_/Si toward a TEM grid. The layer-like 1T-FeCl_2_ synthesized by this method exhibited a triangular symmetric structure, as displayed in Figure 8A (atomic model schematic). This triangular symmetric structure imparted a structural property of semi-hexagonal domains to 1T-FeCl_2_ (Figures 8B–8D) and led to a very flat surface. The crystal thickness was approximately 8.6 nm (Figure 8D), demonstrating quasi-2D characteristics. Attributed to the complete water and oxygen-isolated environment provided by GIS, it is the first time the atomic structure of the very sensitive 2D magnetic transition metal dihalide has been observed. This novel stacking structure in 2D FeCl_2_ differs significantly from traditional understandings. Our results demonstrate that the GIS can serve as a platform for exploring new structures and novel physical properties of sensitive materials. The high-resolution HAADF-STEM image of FeCl_2_ (Figure 8E) revealed high crystallinity, namely, its good crystal quality. The corresponding atomic-scale HAADF-STEM images and the FFT patterns are presented in Figures 8F–8H. The atomic arrangements and lattice contrasts of FeBr_2_/VCl_2_/VBr_2_ conformed to the expected 1T model. This study provides a feasible method for preparing sensitive 2D magnetic TMDCs FeCl_2_/FeBr_2_/VCl_2_/VBr_2_. The FeCl_2_ flakes underwent degradation within a minute when the as-grown FeCl_2_ flakes were placed in the atmosphere (Figures 3H and 3I), suggesting the extreme sensitivity of these TMDHs. Thus, it is imperative to conduct the entire process, encompassing synthesis, sample preparation, and characterization within the GIS to safeguard the intrinsic structure of these materials from hydrolysis and oxidation.

### Beyond 2D: Low-dimensional sensitive materials

The widespread application of glovebox-interconnected systems in studying sensitive 2D materials has considerably assisted material scientists. These systems are not limited to the characterization of single-layer 2D materials while allowing for the preservation of sensitive 2D material heterostructures and low-dimensional systems under glovebox protection. In addition to its crucial role in investigating 2D sensitive materials, GIS has also provided remarkable assistance in researching other air-sensitive materials, demonstrating its broad applicability in handling such materials.

In the field of topological quantum materials, the tetradymite-type MnBi_2_Te_4_ compound was recently discovered as an intrinsic antiferromagnetic topological insulator (AFM) in the A-type AFM ground state with out-of-plane magnetic moments, indicating unique antiferromagnetic properties and topologically protected surface states.^100^^,^^101^^,^^102^ However, the surface of MnBi_2_Te_4_ is highly sensitive; it readily reacts with oxygen and water molecules when exposed to air, resulting in surface oxidation or hydrolysis reactions. This surface degradation further damages the material’s structure, forms an oxide layer, and alters its electronic properties. Therefore, inert gas protection is essential to maintain the stability and performance of 2D MnBi_2_Te_4_.

**Figure 9:**
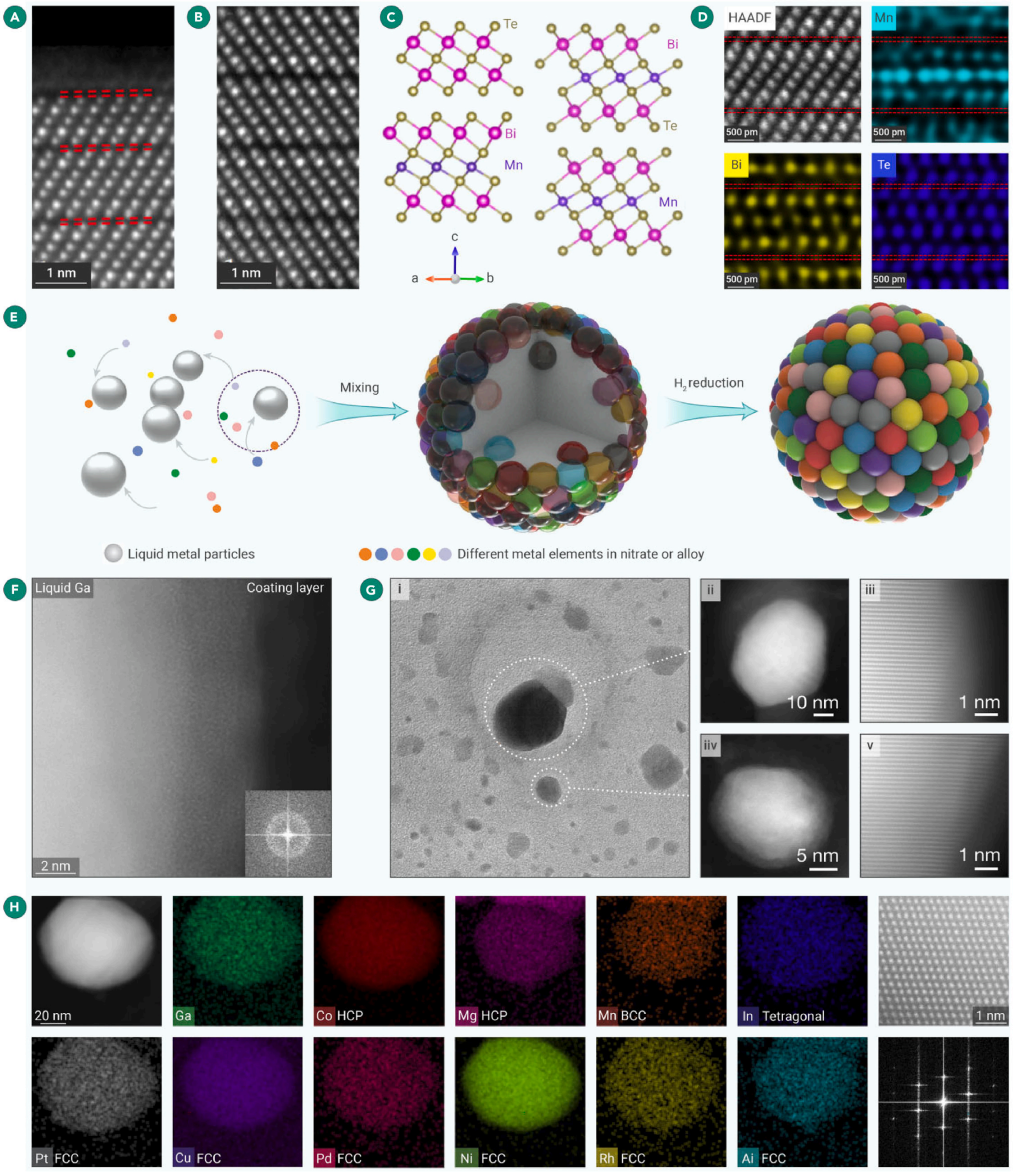
Glovebox protection in the characterization of 3D materials (A) Surface-resolved HAADF-STEM image. Double-layer (DL) crystalline/amorphous structure of the quintuple-layer (QL) MnBi_2_Te_4_ is observed on the surface instead of the ideal seven-layer (SL) structure. The arrow highlights crystalline DL islands on the surface.^103^ (B) Atomic-resolution HAADF-STEM image in the bulk.^103^ (C) Corresponding atomic models for the images.^103^ (D) Atomic-resolution HAADF-STEM image of the SL structure in MnBi_2_Te_4_ and the corresponding EDS mapping of Mn, Bi, and Te elements. The red dashed line highlights a single SL structure.^103^ (E) Schematic for the synthesis of HEA-NPs. (F) HAADF-STEM image for the GaFeMnNiCu HEA precursor. The FFT pattern (inset) corresponded to the amorphous coating layer. (G) The corresponding STEM characterization of the GaFeMnNiCu NPs after cooling down to room temperature. The magnified HAADF-STEM images prove that the NPs become crystalized through the *in situ* experiment. (H) STEM elemental maps, the HAADF-STEM image, and the FFT pattern of an individual undecimal (GaCuPdNiMnAlInRhPtCoMg) HEA-NP, showing the formation of a solid solution structure.

Hou et al. conducted in-depth research on the surface structure of few-layer MnBi_2_Te_4_ and explained the discrepancies between theoretical expectations and experimental results in MnBi_2_Te_4_.^103^ Previously, theoretical predictions and experimental results declared a sizable magnetic gap at the surface of bulk MnBi_2_Te_4_. In contrast, an unambiguously gapless Dirac cone at the (00l) surface of the MnBi_2_Te_4_ crystal was discovered by high-resolution angle-resolved photoemission spectroscopy. Under the protection of an inert gas atmosphere provided by GIS, HAADF-STEM images demonstrated significant structure differences between the surface MnBi_2_Te_4_ and bulk MnBi_2_Te_4_ (Figures 9A and 9B). Compared with the bulk structure, the surface MnBi_2_Te_4_ consists of only five atomic layers and forms a new structure named the quintuple layer (QL). In addition, a layer of amorphous phase was observed above the QL structure, separated from the QL surface, forming a visible vdW gap, as indicated by the red dashed line in Figure 9A. The bulk MnBi_2_Te_4_ exhibited a seven-layer (SL) structure consistent with previous literature reports with the atomic models of the surface MnBi_2_Te_4_ and bulk MnBi_2_Te_4_ (Figure 9C).^104^^,^^105^ Atomic-resolution EDS mapping (Figure 9D) showcased distinctly mixed signals (Bi and Mn) in all Bi and Mn atomic columns, with no corresponding signal detected in the Te layers, revealing an intra-layer exchange between Bi and Mn atoms. The delicate, pristine surface structure was preserved by introducing inert gas protection provided by GIS, contributing to accurately elucidating the structure-property relationship in MnBi_2_Te_4_. This discovery offers a more comprehensive understanding of the antiferromagnetic properties and anomalous quantum states of few-layer MnBi_2_Te_4_ devices, assisting in better investigating surface structure-related measurements and exotic quantum phenomena.

Cao et al. developed a mild synthesis method using liquid metal (Ga-assisted) medium to produce High-entropy alloy nanoparticles (HEA-NPs),^106^ as illustrated in the schematic in Figure 9E. This synthetic approach enables the preparation of HEA-NPs containing up to 11 different elements, overcoming the incompatibility within alloy systems. However, the surfaces of the formed alloy nanoparticles have many unsaturated dangling bonds due to multiple elements. Thus, HEA-NPs demonstrate high surface activity, and the particles adsorb amorphous hydrocarbons when exposed to air, leading to their weakened catalytic or mechanical performance.

Considering this issue, the GIS is employed to conduct the synthesis process under the protection of an inert gas atmosphere. In this case, the synthesis and characterization processes are contamination- and oxidation-free. TEM images (Figures 9F and 9G) reveal that, after HEA-NP fabrication, a clean surface appeared without any adsorption, and HADAF-STEM images showcase the perfect crystalline structure of HEA-NPs. EDS was performed to determine the distribution of different elements within the nanoparticles and confirm the uniform distribution of all the mixed elements. The inert gas atmosphere provided by GIS significantly curtailed the formation of absorbed contamination and surface oxidation, highlighting the versatility of the glovebox interconnect system in the research of air-sensitive materials.

## Conclusion

Regarding the main breakthrough of our GIS, the GIS has achieved complete water and oxygen isolation throughout the entirely experimental streamline, by providing a highly integrated inert gas atmosphere-protected system. This highlights that the GIS enables one-stop sample preparation, device fabrication, functionalization, and characterization of water-sensitive 2D materials, which are difficult to achieve through the current glovebox or vacuum system. In addition to providing a highly integrated experimental platform that completely isolates water and oxygen, the GIS has demonstrated significant scientific value in various perspectives: (1) considering the particular need and challenges of TEM sample fabrication and sample interaction in the inert gas environment and (2) supporting the further exploration of sensitive 2D materials and future physical exploration under inert environment.

First, the GIS is the first attempt to unify TEM with an inert gas environment in an integrated system. During the construction and operation of the GIS, our research group investigated the environmental interaction with advanced instrumentation. The environmental interaction with advanced instrumentation, which has never been considered before in commercialized glovebox systems, was explored during the construction and operation of the GIS. Moreover, as the CVD unit was integrated into the GIS, this technical innovation allowed the investigation of the growth conditions of air-sensitive materials under inert atmosphere protection. In this complete water- and oxygen-free environment, high-quality 2D transition metal dihalides and dichalcogenides have been successfully synthesized.

In addition, the GIS enabled researchers to deeply reveal the degradation processes of sensitive samples in different environments and provided unique solutions for preserving the intrinsic structure of these samples. The comparison of the results of sensitive samples prepared in air and the glovebox enlightened how oxygen and water in air damage the intrinsic structure. The electron microscopy data suggest that samples prepared cleanly in the GIS exhibited enhanced resistance to beam damage, offering new approaches to improve the irradiation tolerance of electron beam-sensitive samples. In conclusion, the GIS significantly strengthened the scientific study of air-sensitive materials by providing a stable and controlled environment for their synthesis, handling, and characterization. It is crucial for advancing understanding and unlocking their potential in various technological applications.

Although the GIS provides an optimal solution for characterizing sensitive 2D materials, there are still numerous challenges during sample preparation through GIS at this stage. Static charge accumulation occurred within the GIS enclosure owing to the isolation from water. The static eliminators cannot fully cover the whole region, leading to the potential breakdown of samples during the device transport test. In addition, the limitations of the glovebox’s circulation system make it challenging to filter out organic solvent vapors and dust completely. The atmosphere within the enclosure gradually deteriorated, resulting in unexpected sample contamination. Furthermore, a particular sealed container is required as the samples prepared in the glovebox need to be further transferred to the PPMS, STM, and other functional GIS units that will be built later. Introducing the sealed container is part of our glovebox interconnect strategies to ensure that our samples are compatible with other research groups' glovebox systems. Consequently, our samples can be exchanged with other research groups worldwide by the glovebox systems, facilitating collaboration in conducting complex experiments.

Meanwhile, the contamination must be further curtailed to further reveal the intrinsic structure of the sensitive 2D materials. Hence, pollutants such as plastics, dust, and organic solvents should be restricted. Specifically, plasticizers (such as storage boxes, plastic bags, and wires) that gradually release contaminants into the surrounding environment should be replaced with metal alternatives or encased to prevent releasing contamination. For dust contamination introduced into the environment, it is necessary to optimize the circulation path of gases within the glovebox to ensure that dust particles can adequately pass through the filtration units rather than settling on the glovebox’s bottom and walls. Regular cleaning and wiping to minimize residual dust are also imperative. Regarding organic solvents, the initial steps consist of maximizing the elimination of water and oxygen in the pre-chamber, and minimizing their usage should be addressed. During the transfer process, any emitted organic vapors can be effectively absorbed by increasing the airflow circulation and placing molecular sieves near the solvents.

Another method to preserve material characteristics and minimize contamination during sample transfer is integrating a low-temperature transfer system into the GIS, which can be considered a promising solution. Traditional transfer methods, which require heating (100°C–200°C) to control the viscosity of the organic adhesive layer, inevitably lead to the volatilization of organics and thermal damage to the samples. Thermal damage and organic contamination can be effectively eliminated by combining a low-temperature transfer platform (semiconductor cooling or liquid nitrogen cooling) with adhesion organic layers that can be used for transferring at low temperatures. In addition, the integrated GIS, paired with low-temperature sample holders and ultra-low-temperature electron microscopes, may provide new approaches for exploring superconducting materials, all of which are performed at low temperatures without exposure to air.

Moreover, transferring water- and oxygen-sensitive samples from the glovebox to the TEM requires vacuum holders to prevent damage. In this system, samples are pre-loaded onto vacuum holders within the glovebox, and then the holders are operated to seal the samples within their vacuum chambers. However, current vacuum holders adopt simple mechanical sealing methods, making it difficult to completely isolate water and oxygen. Inevitably, air leakage occurs, impeding TEM characterization of sensitive materials (especially for monolayers, damage is severe).

Two solutions were proposed to address this issue. First, the sealing ability of the vacuum holders was enhanced. In detail, sample sealing during the insertion and retraction relied on rubber rings and vacuum grease attached to the metal shaft of the holders, whereas this had structural limitations resulting in unavoidable leakage. Therefore, a new design for vacuum holders with enhanced sealing ability was required. The second solution was directly integrating an electron microscope into the glovebox system. Specifically, the GIS was designed to connect directly with the sample loading zone of the electron microscope, allowing direct sample transfer from the glovebox to the microscope. This approach, however, required additional design efforts, significant funding, and considerable lab space. When connected, the positive pressure of the inert gas atmosphere and charge and contaminants accumulation in the glovebox may potentially cause damage to the electron microscope. Despite these challenges, it is optimistically anticipated that more refined solutions will address these issues shortly.

The GIS must accommodate more advanced instrumentation within its limited space to satisfy the growing demands of scientific research. This necessitated a highly space-efficient design that prioritized operational convenience and experimental flexibility. This requires greater integration and automation across all units within the system. Key upgrades involved incorporating a fully automated transfer platform, optical microscope systems, and transport device fabrication units.

Furthermore, integrating artificial intelligence technology significantly improved the GIS by enabling an automated sample synthesis-characterization pipeline. This pipeline can intelligently select suitable characterization techniques following the specific properties of sensitive materials, contributing to improving research efficiency and minimizing sample damage. These advancements will enable the GIS to more effectively support high-precision and high-sensitivity material studies and offer a versatile solution for investigating the structure-property relationships in materials.

## Acknowledgments

This work was supported by the National Key Basic Research and Development Program of China, China (No. 2024YFA1409100). We acknowledges support by the National Natural Science Foundation of China, China (Nos. 52473302 and 12461160252); 10.13039/100012541Guangdong Innovative and Entrepreneurial Research Team Program, China (No. 2019ZT08C044), Guangdong Basic Science Foundation, China (2023B1515120039), Shenzhen Science and Technology Program, China (No. 20200925161102001), the 10.13039/501100010877Science, Technology and Innovation Commission of Shenzhen Municipality, China (No. ZDSYS20190902092905285), and Quantum Science Strategic Special Project from the Quantum Science Center of Guangdong-Hong Kong-Macao Greater Bay Area, China (No. GDZX2301006).

## Author contributions

J.L. supervised and revised the manuscript. Q.Y., X.L., and L.Z. wrote and edited the manuscript. G.W., Z.G., K.N., S.J., and F.H. provided supporting data and parameters of the glovebox system. All authors contributed to the article and approved the submitted version.

## Declaration of interests

The authors declare no competing interests.

## Lead contact website

https://www.sustech.edu.cn/zh/faculties/linjunhao.html

## References

[1] Novoselov K.S., Geim A.K., Morozov S.V. (2004). Electric Field Effect in Atomically Thin Carbon Films. Science.

[2] Lin Z., Carvalho B.R., Kahn E. (2016). Defect engineering of two-dimensional transition metal dichalcogenides. 2D Mater..

[3] Zou X., Xu Y., Duan W. (2021). 2D materials: rising star for future applications. Innovation.

[4] Xu L., Iqbal R., Wang Y. (2024). Emerging two-dimensional materials: Synthesis, physical properties, and application for catalysis in energy conversion and storage. Innovat. Mater..

[5] Naguib M., Kurtoglu M., Presser V. (2011). Two-Dimensional Nanocrystals Produced by Exfoliation of Ti_3_AlC_2_. Adv. Mater..

[6] Dey C., Kundu T., Biswal B.P. (2014). Crystalline metal-organic frameworks (MOFs): synthesis, structure and function. Acta Crystallogr. B Struct. Sci. Cryst. Eng. Mater..

[7] Choi H.S., Lin J., Wang G. (2024). Molecularly thin, two-dimensional all-organic perovskites. Science.

[8] Ding S.-Y., Wang W. (2013). Covalent organic frameworks (COFs): from design to applications. Chem. Soc. Rev..

[9] Geim A.K., Grigorieva I.V. (2013). Van der Waals heterostructures. Nature.

[10] Zhou J., Zhu C., Zhou Y. (2023). Composition and phase engineering of metal chalcogenides and phosphorous chalcogenides. Nat. Mater..

[11] Abdelwahab I., Tilmann B., Wu Y. (2022). Giant second-harmonic generation in ferroelectric NbOI_2_. Nat. Photonics.

[12] Manzeli S., Ovchinnikov D., Pasquier D. (2017). 2D transition metal dichalcogenides. Nat. Rev. Mater..

[13] Zhu J., Hu Z., Guo S. (2023). Non-epitaxial growth of highly oriented transition metal dichalcogenides with density-controlled twin boundaries. Innovation.

[14] Kulish V.V., Huang W. (2017). Single-layer metal halides MX_2_ (X = Cl, Br, I): stability and tunable magnetism from first principles and Monte Carlo simulations. J. Mater. Chem. C Mater..

[15] Jiang S., Wang G., Deng H. (2023). General Synthesis of 2D Magnetic Transition Metal Dihalides via Trihalide Reduction. ACS Nano.

[16] Botana A.S., Norman M.R. (2019). Electronic structure and magnetism of transition metal dihalides: Bulk to monolayer. Phys. Rev. Mater..

[17] Zhou X., Jiang T., Tao Y. (2024). Evidence of Ferromagnetism and Ultrafast Dynamics of Demagnetization in an Epitaxial FeCl_2_ Monolayer. ACS Nano.

[18] Rhodes D., Chae S.H., Ribeiro-Palau R. (2019). Disorder in van der Waals heterostructures of 2D materials. Nat. Mater..

[19] Mannix A.J., Kiraly B., Hersam M.C. (2017). Synthesis and chemistry of elemental 2D materials. Nat. Rev. Chem.

[20] Frisenda R., Navarro-Moratalla E., Gant P. (2018). Recent progress in the assembly of nanodevices and van der Waals heterostructures by deterministic placement of 2D materials. Chem. Soc. Rev..

[21] Ruska E. (1987). The development of the electron microscope and of electron microscopy. Rev. Mod. Phys..

[22] Hall C. (1953).

[23] Lin G., Shiyu W., Qinghua Z. (2024). Direct observation of charge density and electronic polarization in fluorite ferroelectrics by 4D-STEM. Innovat. Mater..

[24] Jesson D.E., Pennycook S.J. (1995). Incoherent imaging of crystals using thermally scattered electrons. Proc. Roy. Soc. Lond. Math. Phys. Sci..

[25] Crewe A.V., Wall J., Langmore J. (1970). Visibility of Single Atoms. Science.

[26] Pennycook S.J., Nellist P.D. (2011).

[27] Nellist P.D., Pennycook S.J., Hawkes P.W. (2000). Advances in Imaging and Electron Physics.

[28] Pennycook S.J., Boatner L.A. (1988). Chemically sensitive structure-imaging with a scanning transmission electron microscope. Nature.

[29] Pennycook S.J. (1989). Z-contrast stem for materials science. Ultramicroscopy.

[30] Pennycook S.J., Jesson D.E. (1991). High-resolution Z-contrast imaging of crystals. Ultramicroscopy.

[31] Wang L., Meric I., Huang P.Y. (2013). One-Dimensional Electrical Contact to a Two-Dimensional Material. Science.

[32] Ma X., Liu Q., Xu D. (2017). Capillary-Force-Assisted Clean-Stamp Transfer of Two-Dimensional Materials. Nano Lett..

[33] Ali U., Karim K.J.B.A., Buang N.A. (2015). A Review of the Properties and Applications of Poly (Methyl Methacrylate) (PMMA). Polym. Rev..

[34] Dong W., Dai Z., Liu L. (2024). Toward Clean 2D Materials and Devices: Recent Progress in Transfer and Cleaning Methods. Adv. Mater..

[35] Island J.O., Steele G.A., van der Zant H.S.J. (2015). Environmental instability of few-layer black phosphorus. 2D Mater..

[36] Mirabelli G., McGeough C., Schmidt M. (2016). Air sensitivity of MoS_2_, MoSe_2_, MoTe_2_, HfS_2_, and HfSe_2_. J. Appl. Phys..

[37] Cao Y., Mishchenko A., Yu G.L. (2015). Quality Heterostructures from Two-Dimensional Crystals Unstable in Air by Their Assembly in Inert Atmosphere. Nano Lett..

[38] Zhao S., Wang E., Üzer E.A. (2021). Anisotropic moiré optical transitions in twisted monolayer/bilayer phosphorene heterostructures. Nat. Commun..

[39] Lin Y.-C., Matsumoto R., Liu Q. (2024). Alkali metal bilayer intercalation in graphene. Nat. Commun..

[40] Wang H., Huang X., Lin J. (2017). High-quality monolayer superconductor NbSe_2_ grown by chemical vapour deposition. Nat. Commun..

[41] Lin Y.-C., Motoyama A., Kretschmer S. (2021). Polymorphic Phases of Metal Chlorides in the Confined 2D Space of Bilayer Graphene. Adv. Mater..

[42] Gray M.J., Kumar N., O’Connor R. (2020). A cleanroom in a glovebox. Rev. Sci. Instrum..

[43] Dobkin D., Zuraw M.K. (2003).

[44] Niu K., Weng M., Li S. (2021). Direct Visualization of Large-Scale Intrinsic Atomic Lattice Structure and Its Collective Anisotropy in Air-Sensitive Monolayer 1T’- WTe_2_. Adv. Sci..

[45] Liao L., Bai J., Qu Y. (2010). High-κ oxide nanoribbons as gate dielectrics for high mobility top-gated graphene transistors. Proc. Natl. Acad. Sci. USA.

[46] Liao L., Lin Y.-C., Bao M. (2010). High-speed graphene transistors with a self-aligned nanowire gate. Nature.

[47] Huang B., Clark G., Navarro-Moratalla E. (2017). Layer-dependent ferromagnetism in a van der Waals crystal down to the monolayer limit. Nature.

[48] Zhang Z., Shang J., Jiang C. (2019). Direct Photoluminescence Probing of Ferromagnetism in Monolayer Two-Dimensional CrBr3. Nano Lett..

[49] McGuire M.A., Clark G., Kc S. (2017). Magnetic behavior and spin-lattice coupling in cleavable van der Waals layered CrCl_3_ crystals. Phys. Rev. Mater..

[50] Kong T., Stolze K., Timmons E.I. (2019). VI3—a New Layered Ferromagnetic Semiconductor. Adv. Mater..

[51] Zhou X., Brzostowski B., Durajski A. (2020). Atomically Thin 1T-FeCl_2_ Grown by Molecular-Beam Epitaxy. J. Phys. Chem. C.

[52] Bikaljević D., González-Orellana C., Peña-Díaz M. (2021). Noncollinear Magnetic Order in Two-Dimensional NiBr_2_ Films Grown on Au(111). ACS Nano.

[53] Mannix A.J., Ye A., Sung S.H. (2022). Robotic four-dimensional pixel assembly of van der Waals solids. Nat. Nanotechnol..

[54] Kang K., Lee K.-H., Han Y. (2017). Layer-by-layer assembly of two-dimensional materials into wafer-scale heterostructures. Nature.

[55] Manzeli S., Ovchinnikov D., Pasquier D. (2017). 2D transition metal dichalcogenides. Nat. Rev. Mater..

[56] Mak K.F., Shan J. (2016). Photonics and optoelectronics of 2D semiconductor transition metal dichalcogenides. Nat. Photonics.

[57] Chhowalla M., Shin H.S., Eda G. (2013). The chemistry of two-dimensional layered transition metal dichalcogenide nanosheets. Nat. Chem..

[58] Sierra J.F., Fabian J., Kawakami R.K. (2021). Van der Waals heterostructures for spintronics and opto-spintronics. Nat. Nanotechnol..

[59] Wang Q.H., Kalantar-Zadeh K., Kis A. (2012). Electronics and optoelectronics of two-dimensional transition metal dichalcogenides. Nat. Nanotechnol..

[60] Tahir M.B., Fatima U. (2022). Recent trends and emerging challenges in two-dimensional materials for energy harvesting and storage applications. Energy Storage.

[61] Lee M.H., Wu W. (2022). 2D Materials for Wearable Energy Harvesting. Adv. Mater. Technol..

[62] Piacentini A., Daus A., Wang Z. (2023). Potential of Transition Metal Dichalcogenide Transistors for Flexible Electronics Applications. Adv. Electron. Mater..

[63] Jiang J., Liu Z.K., Sun Y. (2017). Signature of type-II Weyl semimetal phase in MoTe_2_. Nat. Commun..

[64] Ruppert C., Aslan B., Heinz T.F. (2014). Optical Properties and Band Gap of Single- and Few-Layer MoTe_2_ Crystals. Nano Lett..

[65] Ali M.N., Xiong J., Flynn S. (2014). Large, non-saturating magnetoresistance in WTe_2_. Nature.

[66] Kang K., Li T., Sohn E. (2019). Nonlinear anomalous Hall effect in few-layer WTe_2_. Nat. Mater..

[67] Xu X., Pan Y., Liu S. (2021). Seeded 2D epitaxy of large-area single-crystal films of the van der Waals semiconductor 2H MoTe_2_. Science.

[68] Cheon Y., Lim S.Y., Kim K. (2021). Structural Phase Transition and Interlayer Coupling in Few-Layer 1T′ and Td MoTe_2_. ACS Nano.

[69] Kim H.-J., Kang S.-H., Hamada I. (2017). Origins of the structural phase transitions in MoTe_2_ and WTe_2_. Phys. Rev. B.

[70] Guo Z., Wang L., Han M. (2022). One-Step Growth of Bilayer 2H–1T′ MoTe_2_ van der Waals Heterostructures with Interlayer-Coupled Resonant Phonon Vibration. ACS Nano.

[71] Peng L., Yuan Y., Li G. (2017). Observation of topological states residing at step edges of WTe_2_. Nat. Commun..

[72] Soluyanov A.A., Gresch D., Wang Z. (2015). Type-II Weyl semimetals. Nature.

[73] Qi Y., Naumov P.G., Ali M.N. (2016). Superconductivity in Weyl semimetal candidate MoTe2. Nat. Commun..

[74] Torun E., Sahin H., Cahangirov S. (2016). Anisotropic electronic, mechanical, and optical properties of monolayer WTe_2_. J. Appl. Phys..

[75] Frenzel A.J., Homes C.C., Gibson Q.D. (2017). Anisotropic electrodynamics of type-II Weyl semimetal candidate WTe_2_. Phys. Rev. B.

[76] Thirupathaiah S., Jha R., Pal B. (2017). MoTe_2_: An uncompensated semimetal with extremely large magnetoresistance. Phys. Rev. B.

[77] Wang W., Kim S., Liu M. (2020). Evidence for an edge supercurrent in the Weyl superconductor MoTe_2_. Science.

[78] Song T., Jia Y., Yu G. (2024). Unconventional superconducting quantum criticality in monolayer WTe_2_. Nat. Phys..

[79] Hsu Y.-T., Cole W.S., Zhang R.-X. (2020). Inversion-Protected Higher-Order Topological Superconductivity in Monolayer WTe_2_. Phys. Rev. Lett..

[80] Guo Z., Han M., Zeng S. (2024). Intrinsic Grain Boundary Structure and Enhanced Defect States in Air-Sensitive Polycrystalline 1T’-WTe_2_ Monolayer. Adv. Mater..

[81] Pace S., Martini L., Convertino D. (2021). Synthesis of Large-Scale Monolayer 1T′-MoTe_2_ and Its Stabilization via Scalable hBN Encapsulation. ACS Nano.

[82] Naylor C.H., Parkin W.M., Gao Z. (2017). Large-area synthesis of high-quality monolayer 1T’-WTe_2_ flakes. 2D Mater..

[83] Elibol K., Susi T., Argentero G. (2018). Atomic Structure of Intrinsic and Electron-Irradiation-Induced Defects in MoTe_2_. Chem. Mater..

[84] Hart J.L., Bhatt L., Zhu Y. (2023). Emergent layer stacking arrangements in c-axis confined MoTe_2_. Nat. Commun..

[85] Yuan F., Jia Y., Cheng G. (2023). Atomic Resolution Imaging of Highly Air-Sensitive Monolayer and Twisted-Bilayer WTe_2_. Nano Lett..

[86] Zhou Z., Hou F., Huang X. (2023). Stack growth of wafer-scale van der Waals superconductor heterostructures. Nature.

[87] Gibertini M., Koperski M., Morpurgo A.F. (2019). Magnetic 2D materials and heterostructures. Nat. Nanotechnol..

[88] Meng L., Zhou Z., Xu M. (2021). Anomalous thickness dependence of Curie temperature in air-stable two-dimensional ferromagnetic 1T-CrTe_2_ grown by chemical vapor deposition. Nat. Commun..

[89] Zhang X., Lu Q., Liu W. (2021). Room-temperature intrinsic ferromagnetism in epitaxial CrTe_2_ ultrathin films. Nat. Commun..

[90] Xian J.-J., Wang C., Nie J.-H. (2022). Spin mapping of intralayer antiferromagnetism and field-induced spin reorientation in monolayer CrTe_2_. Nat. Commun..

[91] Zhong Y., Peng C., Huang H. (2023). From Stoner to local moment magnetism in atomically thin Cr_2_Te_3_. Nat. Commun..

[92] Liu X., Gebredingle Y., Guo X. (2024). Thickness-Dependent Crystal Structure, Synthesis, and Magnetism of Thin Film Chromium Chalcogenides: A Review. Adv. Funct. Mater..

[93] Chi H., Ou Y., Eldred T.B. (2023). Strain-tunable Berry curvature in quasi-two-dimensional chromium telluride. Nat. Commun..

[94] Huang M., Ma Z., Wang S. (2021). Significant perpendicular magnetic anisotropy in room-temperature layered ferromagnet of Cr-intercalated CrTe_2_. 2D Mater..

[95] Bian M., Kamenskii A.N., Han M. (2021). Covalent 2D Cr_2_Te_3_ ferromagnet. Mater. Res. Lett..

[96] Bian M., Zhu L., Wang X. (2022). Dative Epitaxy of Commensurate Monocrystalline Covalent van der Waals Moiré Supercrystal. Adv. Mater..

[97] Tang B., Wang X., Han M. (2022). Phase engineering of Cr_5_Te_8_ with colossal anomalous Hall effect. Nat. Electron..

[98] Niu K., Qiu G., Wang C. (2023). Self-Intercalated Magnetic Heterostructures in 2D Chromium Telluride. Adv. Funct. Mater..

[99] Han X., You J.-Y., Wu S. (2023). Atomically Unveiling an Atlas of Polytypes in Transition-Metal Trihalides. J. Am. Chem. Soc..

[100] Deng Y., Yu Y., Shi M.Z. (2020). Quantum anomalous Hall effect in intrinsic magnetic topological insulator MnBi_2_Te_4_. Science.

[101] Sass P.M., Kim J., Vanderbilt D. (2020). Robust A-Type Order and Spin-Flop Transition on the Surface of the Antiferromagnetic Topological Insulator MnBi_2_Te_4_. Phys. Rev. Lett..

[102] Li J., Li Y., Du S. (2019). Intrinsic magnetic topological insulators in van der Waals layered MnBi_2_Te_4_-family materials. Sci. Adv..

[103] Hou F., Yao Q., Zhou C.-S. (2020). Te-Vacancy-Induced Surface Collapse and Reconstruction in Antiferromagnetic Topological Insulator MnBi_2_Te_4_. ACS Nano.

[104] Gong Y., Guo J., Li J. (2019). Experimental Realization of an Intrinsic Magnetic Topological Insulator. Chin. Phys. Lett..

[105] Li H., Liu S., Liu C. (2020). Antiferromagnetic topological insulator MnBi2Te4: synthesis and magnetic properties. Phys. Chem. Chem. Phys..

[106] Cao G., Liang J., Guo Z. (2023). Liquid metal for high-entropy alloy nanoparticles synthesis. Nature.

